# Toxin–antitoxins and sigma factors may optimize the fitness of free-living bacteria throughout the life cycle via an integrated nutrient-responsive cybernetic system

**DOI:** 10.1098/rsif.2025.0185

**Published:** 2025-09-17

**Authors:** Stephen J. Knabel, Ramaswamy Anantheswaran, Aubrey Mendonca, Wei Zhang

**Affiliations:** ^1^ Department of Food Science, Pennsylvania State University, University Park, PA, USA; ^2^ Department of Food Science and Human Nutrition, Iowa State University, Ames, IA, USA; ^3^ Department of Food Science & Nutrition, Illinois Institute of Technology, Bedford Park, IL, USA

**Keywords:** toxin–antitoxins, sigma factors, nutrient-responsive, cybernetic system, life cycle, fitness

## Abstract

Toxin–antitoxin systems (TASs) are ubiquitous in the chromosomes of free-living bacteria, yet their primary biological function remains poorly understood. Bacteria reproduce exponentially via 2^*n*^ growth kinetics and thus must respond to changing nutrient availability to reproduce rapidly during short periods of feast and survive during long periods of famine. Type II TASs represent stable enzyme-unstable inhibitor systems that are regulated by reversible competitive inhibition, which allows them to efficiently produce pleiotropic effects on prokaryotic cells in a continuous (analogue) manner due to varying concentrations of free toxin throughout the life cycle. A nutrient-responsive cybernetic system (NRCS) model is proposed where intracellular nutrient concentration feeds back to control the emergent properties of growth, death and growth/death arrest, which results in a novel fitness strategy termed K Sensing and Control. When nutrients become limiting, alternative general stress response sigma factors Ϭ^S^ and Ϭ^B^ regulate the expression of hundreds of genes that may control the transformation of vegetative bacteria into coccoid, stress-tolerant ‘motherspores’. An integrated NRCS model is presented that shows how TASs and sigma factors may work in concert to efficiently regulate population dynamics, cellular physiology and cellular differentiation throughout the life cycle, which optimizes the biological fitness of free-living bacteria.

## 1. Chromosomal toxin–antitoxin systems are ubiquitous; however, their main biological function remains unclear

Toxin–antidote elements are common across the tree of life [1]. In bacteria, toxin–antitoxin systems (TASs) were first discovered on plasmids and shown to play a role in plasmid maintenance and stabilization [2], as cells would be killed if the plasmid was lost, a process termed post-segregational killing (PSK), which results in plasmid addiction [3]. They were subsequently shown to be ubiquitous in the chromosomes of free-living bacteria [4], which are defined as those bacteria able to live independently of other organisms. The widespread presence of these modules, many of which appear to have been acquired through horizontal gene transfer, has driven extensive research over the past several decades, aimed at better understanding their possible physiological roles and characterizing the conditions leading to their activation [5]. While the presence of TASs in plasmids allows them to propagate selfishly, the abundance of TASs in the chromosomes of free-living bacteria suggests that they may serve a function of basic biological importance to these highly diverse organisms [1,6]. However, the main biological function of chromosomal TASs continues to be described by various authors as ambiguous, challenging, contradictory, a conundrum, difficult to determine, elusive, enigmatic, highly debated, highly speculative, lacking a broad consensus, mysterious, obscure, a paradox, poorly understood, a puzzle, a riddle, speculative, unanswered, unclear, unexplained, unknown, unresolved and unsolved [6–26]. The following numerous and sometimes conflicting hypotheses have been suggested as possible biological functions for chromosomal TASs: (i) junk DNA; (ii) selfish elements; (iii) stabilization of mobile genomic elements; (iv) anti-addiction; (v) gene regulation; (vi) growth control; (vii) stress response; (viii) growth arrest/dormancy; (ix) programmed cell death (PCD); (x) persister cell formation; (xi) biofilm formation; (xii) phage defence via a process known as abortive infection (Abi); and (xiii) virulence/pathogenesis [1,19,22,26–31]. However, the hypothesis that chromosomal TASs may optimize the fitness of free-living bacteria by working in concert with sigma factors as an integrated nutrient-responsive cybernetic system (NRCS) to efficiently regulate population dynamics, cellular physiology and cellular differentiation throughout the life cycle has not yet been proposed.

## 2. Free-living bacteria must efficiently transition between feast and famine modes of existence

Bacteria reproduce asexually via binary fission, which allows populations to increase exponentially via 2^*n*^ growth kinetics. To get an appreciation for the tremendous reproductive potential of free-living bacteria, it has been calculated that if one *Escherichia coli* cell could continue to double constantly every 20 min under ideal conditions, the resultant mass of growth would weigh more than the weight of the Earth within 48 h. Clearly, this does not occur as they would run out of nutrients long before this happens; thus, bacteria spend most of their time in prolonged states of very low metabolic activity with little or no growth [32]. Therefore, a major dilemma facing all free-living bacteria throughout their long evolutionary history has been that they must reproduce as fast as possible to out-compete other bacteria when nutrients are plentiful, but then rapidly switch to a dormant and stress-tolerant state when nutrients become limiting. The ability to sense and respond to fluctuations in environmental nutrient levels is a requisite for life and a central topic of microbiology [33]. One of the most daunting challenges in biology has been elucidating the mechanisms by which bacteria sense and respond to changes in nutrient status [34]. However, scientists have tended to avoid investigating how bacteria survive for long periods of time in dormant and stress-tolerant states; thus, little is known about the fundamental mechanisms that underpin this mode of existence [32]. Patnaik [35] pointed out that the growth and metabolic capabilities of microorganisms depend on their interactions with the environments they inhabit [35]. He reviewed the cybernetic modelling of microbial metabolism; however, he did not mention TASs or how they might be used to efficiently control the population dynamics and cellular physiology of free-living bacteria throughout the life cycle. Ramkrishna [36] and Stebbing [37] argued that many biological organisms, including microorganisms, possess internally programmed cybernetic systems that allow them to make the critical transition between nutrient abundance and nutrient limitation; however, they failed to identify the specific molecular mechanism(s) that enable it [36,37]. Therefore, the question is no longer are prokaryotic populations regulated, but how are they regulated [38]. In this review, a model will be proposed that shows how chromosomal TASs may work in concert with sigma factors as an integrated NRCS to enable free-living bacteria to efficiently cycle between rapid reproduction and long-term survival (delayed reproduction), both of which dramatically enhance biological fitness.

## 3. Summary of literature on chromosomal toxin–antitoxin systems and sigma factors

### 3.1. Types of toxin–antitoxin systems

While most toxin–antitoxin (TA) toxins are proteins (enzymes), there are currently eight known types of TASs based on the nature of the antitoxin (protein or RNA) and the mechanisms by which it inhibits toxin activity [21,22]. TASs belonging to types I and II are widely distributed throughout the prokaryotic world, whereas fewer representatives are known to date for the other classes. Type II TASs, which are ubiquitous in free-living bacteria, consist of protein antitoxins that form stable complexes with their cognate protein toxins (enzymes), typically neutralizing them by blocking the active site [39]. Most type II TASs are kept inactive when excess nutrients are available, due to the overproduction of antitoxins relative to that of toxins during exponential growth [40,41]. This situation is rapidly reversed during stress [42], when translation of new antitoxins slows down or stops, and existing antitoxins are subsequently degraded by cellular proteases, thus releasing free TA toxins [43]. Numerous type II TA toxins possess sequence-specific RNA cleavage (endonuclease) activity, which diminishes protein synthesis and hence cellular metabolism [39,44]. MazF is one of the most widespread and well-characterized type II TA toxins with endonuclease activity and along with its cognate antitoxin MazE was the first TAS to be found in a bacterial chromosome by Metzger *et al*. [45] when they discovered the *relA* locus in *E. coli* [45]. RelA catalyses the synthesis of the alarmone (p)ppGpp, which activates Ϭ^S^, the alternative sigma factor that is the master regulator of the general stress response in Gram-negative bacteria and is encoded by *rpoS* [46]. In *E. coli*, various stresses lead to higher levels of Ϭ^S^ (RpoS) due to its increased translation and decreased degradation [47]. Upon finding *relA* in the *E. coli* chromosome, Metzger *et al*. [45] also found an open reading frame (ORF) in the *relA* operon immediately downstream of the *relA* locus, which was co-transcribed with *relA* [45]. However, at the time of its discovery, the authors did not know what this ORF encoded and thus named it ‘*ma-ze*’, which in Hebrew means ‘What is it?’ [45]. MazE, along with its cognate toxin MazF, is now one of the most widely distributed and well-characterized bacterial TASs [43,48]. Intriguingly, in Gram-positive bacteria, *mazEF* is directly upstream of the *sigB* operon, which encodes the general stress response alternative sigma factor Ϭ^B^. Like co-transcription of *relA* and *mazEF* in Gram-negative bacteria, *sigB* and *mazEF* are co-transcribed in Gram-positive bacteria [49]. Co-transcription of *mazEF* with both *relA* (which activates Ϭ^S^) and *sigB* (which encodes Ϭ^B^) is consistent with MazF working in concert with these general stress response sigma factors to convert free-living bacteria into dormant and stress-tolerant cells for the purpose of long-term survival in nature. Numerous authors have tied activation of various TA toxins to nutrient starvation [19,26,31,39,50–54].

### 3.2. The nature of toxin–antitoxin systems

TA toxins are almost always proteins (enzymes), and their cognate antitoxins are either proteins or RNA. TA toxins are unique among bacterial toxins, in that they remain intracellular and cause toxic effects to the bacterial host cells themselves [21,39,55]. The molecular targets of TA toxins are diverse and include DNA replication, transcription, translation and cell wall synthesis [16,44]. Antitoxins interact with and neutralize their cognate toxins, providing both immunity from the toxin and a means to resume bacterial growth following toxin-induced growth arrest [56]. Toxin and antitoxin genes are often located next to each other on the same operon and thus are typically co-transcribed [25]. Antitoxin genes are frequently located upstream of toxin genes, and antitoxin mRNA is translated more rapidly than toxin mRNA, which together ensures antitoxin genes are expressed more abundantly than toxin genes to neutralize the toxic effect of their cognate toxins under non-stress conditions [52]. However, the activity of TA toxins is unleashed during stress when their cognate unstable antitoxins are degraded and new antitoxins cannot be synthesized [4]. The general mechanism of TA action relies on the differential *in vivo* lifetimes of the stable toxin and unstable antitoxin [16], which are determined by their biophysical properties [14]. For example, many protein antitoxins fall under the category of intrinsically disordered/unstructured proteins [57,58]. Such protein antitoxins typically contain two different domains: (i) the N-terminal domain, which interacts with the DNA promoter-binding region of toxin–antitoxin operons to inhibit their transcription, and (ii) the C-terminal domain, which interacts with the cognate toxin to neutralize its toxicity [14]. The C-terminal domain of protein antitoxins is typically disordered (unfolded), which is critical for the proper functioning of TASs, as it makes protein antitoxins susceptible to rapid degradation by cellular proteases, which subsequently results in the release of their free ‘active’ cognate toxins [14,58–60].

While the intracellular concentration of free toxin is controlled by the intracellular concentration of antitoxin, there are two processes that might determine the intracellular concentration of antitoxin: (i) the rate of antitoxin synthesis and (ii) the rate of antitoxin degradation. Maisonneuve *et al*. [61] proposed an ‘active’ model of TA regulation based on enhanced degradation of antitoxin, in which (p)ppGpp mediates activation of TA modules through a signalling pathway involving inorganic polyphosphate and the Lon protease [61]. However, they retracted this paper as the results were later found to be artefacts of inadvertent lysogenization with the bacteriophage ɸ80. However, other researchers also recently proposed that toxin activation was caused by differential protease activation [18,62–64]. In contrast, Ramisetty [65] concluded that neither (p)ppGpp nor polyP is required for the transcriptional upregulation of the TA module *yefM/yoeB* [65]. This conclusion was consistent with earlier findings that transcriptional upregulation of *relBE* [51] and *mazEF* [66] during Shiga toxins (SHX)-induced starvation is independent of (p)ppGpp but dependent on Lon protease. As a result, Ramisetty [52] proposed a ‘passive translation-responsive’ model for type II TASs in which changes in the transcription of the TA operon, which represents the antitoxin/toxin ratio, are a dynamic function of translation [52]. In that model, it was assumed that the rate of protein antitoxin degradation remains constant, while the rate of antitoxin translation (and thus intracellular concentration of antitoxin) varies according to the growth conditions that influence translation [4,40,52]. Ramisetty [52] concluded that the dynamic nature of this type of TA regulation allows sensing the global translation rate, which usually indicates the nature of the growth conditions and available nutrient resources [52]. Consistent with this model, the concentration of free ‘active’ toxins in the cell is now thought to be mainly controlled through toxin sequestration in toxin–antitoxin complexes of various stoichiometry, rather than by gene regulation [67].

In addition to two mechanisms that could control the intracellular concentration of antitoxins, there are also two mechanisms that could control the generation of free TA toxins: (i) the rate of synthesis of new toxins and (ii) the rate of release of pre-existing toxins from T–A complexes due to the enzymatic degradation of their cognate antitoxins. Brantl & Jahn [13] stated that different TASs appear to use one or the other of these two mechanisms, and a few appear to use both [13]. However, regardless of how TA toxins are generated, it must be remembered that the intracellular concentration of antitoxin is the master controller of the intracellular concentration of free toxin, which ultimately determines cell fate [9].

Lioy *et al*. [68] found that the TA toxin zeta caused pleiotropic effects, including death when the toxin is present at high concentrations and growth arrest when the toxin is present at lower concentrations [68]. Yamaguchi *et al.* proposed that MazF would have pleiotropic effects of growth arrest and death depending on the intracellular concentration of MazF and time of exposure to this TA toxin [44]. Similarly, the TA toxin TisB [69] was also reported to cause pleiotropic effects of both death and growth arrest [70]. Wen & Fozo [71] concluded that the pleiotropic effects of TisB are due to the different ratios of toxin : antitoxin inside the cell: when the T : A ratio is high, death occurs, and when the T : A ratio is lower, growth arrest occurs [71]. Harms *et al*. [16] logically concluded that degradation of antitoxin would change the intracellular T : A ratio, which is consistent with antitoxin concentration being the master controller of free toxin activity and thus cell fate [16]. The above-mentioned pleiotropic effects of free TA toxins based on their intracellular concentration are consistent with the well-known dose–response curve for all types of toxins [72].

Vesper *et al*. [73] demonstrated that *E. coli* MazF generates leaderless mRNAs that can only be translated on stress-induced translation machinery (STM) ribosomes when ACA sites are ‘out-of-frame’ [73,74]. The latter authors demonstrated that leaderless mRNAs, which contain ‘in-frame’ ACA sites, are cleaved by MazF bound to ribosomal protein bS1 and thus are not translated into intact proteins. Interestingly, *E. coli* leaderless *mazE* mRNA contains two ACA sites, both of which are ‘out-of-frame’, and thus would be translated into intact MazE proteins by STM ribosomes. In contrast, *E. coli mazF* mRNA contains four ‘in-frame’ ACA sites, which would be cleaved on STM ribosomes, and thus *mazF* mRNA would not be translated into intact MazF proteins by STM ribosomes. Therefore, post-transcriptional autoregulation of MazF activity via both MazE synthesis and *mazF* mRNA cleavage may allow *E. coli* cells to rapidly exit stress caused by MazF [75].

### 3.3. Toxin–antitoxin systems play a role in persister cell formation

Since their discovery, prokaryotic persister cells continue to be defined as a subpopulation of phenotypic variants that are transiently tolerant to numerous antibiotics [76,77]. The antibiotic tolerance of persister cells is thought to be due to TASs shutting down various cellular metabolisms, which makes them dormant and recalcitrant to the action of antibiotics [78]. Seen in this light, persistence is not so much a ‘type of cell’ [79], but an indirect outcome of metabolic inactivity that leads to cell dormancy. *Mycobacterium tuberculosis* has approximately 90 chromosomal TASs, while non-pathogenic species of *Mycobacterium* have only a few. *Mycobacterium tuberculosis* may need this large number of TASs to shut down numerous types of metabolism in order to remain metabolically inactive and thus latent and undetected by the human immune system for long periods of time [80]. The need to inactivate many different types of metabolism and thus remain metabolically inactive during long-term survival in nature may be the answer to two critical questions posed about chromosomal TASs 15 years ago: why so many, what for? [81].

The role of TASs in persister cell formation was supported by loss of persistence upon deletion of several TA toxin genes such as *yafQ* [82], *mqsR* [83] and *tisB* [80]. However, a subsequent paper by Harms *et al*. no longer supported a role for TA modules in *E. coli* persister cell formation [16]. This conclusion was supported by Goormaghtigh *et al*. [15], who also reported no direct link between induction of TASs and persistence [15]. However, Holden & Errington [17] cautioned against this conclusion, as the analysis of Goormaghtigh *et al.* [15] was based on mid-exponential phase cells in an optimally balanced medium. Harrison *et al*. [82] demonstrated that the TA toxin YafQ was responsible for tolerance to the antibiotics cefazolin and tobramycin when cells were in biofilms, but not when planktonic cells were in the stationary phase [82]. This is consistent with the finding that cells within biofilms experience starvation stress, which is known to trigger persister cell formation [84]. TA toxins are thought to be sequestered in an inactive toxin–antitoxin complex by excess antitoxins when cells are actively reproducing at steady state in the log phase [52,56]. This is consistent with the results of Keren *et al*. [85], who demonstrated that persister cells were not formed when log phase cells were frequently transferred into the same nutrient-rich medium at a constant temperature [85]. Such treatment would keep all cells in an unstressed steady state of growth [86]. Similarly, Dawes & Mandelstam [87] found that *Bacillus subtilis* did not form metabolically inactive endospores, which also meet the criteria of persister cells, when cultured at a maximum rate of steady-state growth in a chemostat [87]. In general, the above-mentioned results support the conclusion that TA toxins increase persistence during nutrient stress by inactivating various types of metabolism; however, few chromosomal TASs decrease persistence when deleted [88], perhaps due to their redundant nature [71]. Many authors now support chromosomal TASs playing a role in persister cell formation upon nutrient depletion [12,17,19,26,28,71].

### 3.4. Debate over chromosomally encoded toxin–antitoxin systems causing programmed cell death

**Figure 1.**
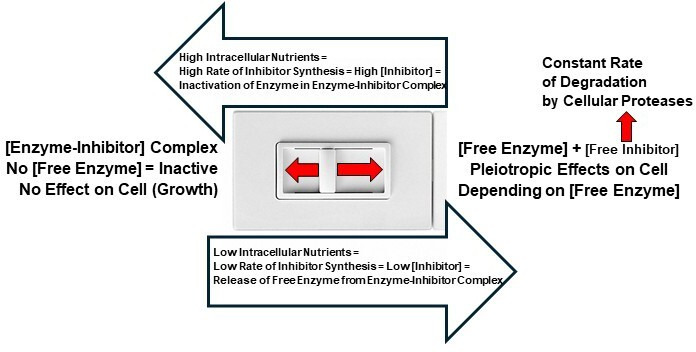
Like a sliding dimmer switch, type II TASs act as enzyme-inhibitor systems that use reversible competitive inhibition via an unstable inhibitor that is degraded at a constant rate to rapidly and efficiently regulate population dynamics and cellular physiology throughout the life cycle in a continuous (analogue) manner based on the concentration of intracellular nutrients. Varying intracellular nutrient concentrations result in varying rates of inhibitor synthesis, which result in varying inhibitor concentrations, which result in varying concentrations of free toxin that have pleiotropic effects on prokaryotic cells (Germination and Growth at low [Free Enzyme] below *K*, PCD at high [Free Enzyme] above *K* and Growth/Death Arrest at constant/moderate [Free Enzyme] at *K*; figure 2).

Since the initial discovery that plasmid-encoded TA toxins play a role in PSK [2], numerous TA genes have been found in the chromosomes of free-living bacteria, where they are active and homologous to plasmid-encoded TA toxins [50], and thus are now thought by many authors to play a role in PCD [55,89–93]. Nieto *et al*. [94] demonstrated that ectopic overexpression of the TA toxin YoeB caused a dramatic loss in the viability of *E. coli*, and Fu *et al*. [95] demonstrated a dramatic loss in the viability of *Staphylococcus aureus* upon ectopic overexpression of *mazF* [94,95]. However, many authors in the field have argued that TA toxins only cause growth arrest, either because new TA toxins are not synthesized at high enough levels *in vivo* to cause PCD [66,94–96] or antitoxins are so tightly bound to toxins that the antitoxin cannot be degraded and thus the toxin cannot be released [22]. However, Bordes & Genevaux [18] suggested that antitoxins would be degraded *in vivo* when they dissociated from toxin–antitoxin complexes, thereby releasing free toxins [18]. Figure 1 shows how TASs may function as stable enzyme-unstable inhibitor systems that use reversible competitive inhibition [97] in a continuous (analogue) manner to efficiently regulate pleiotropic effects throughout the life cycle.

**Figure 2.**
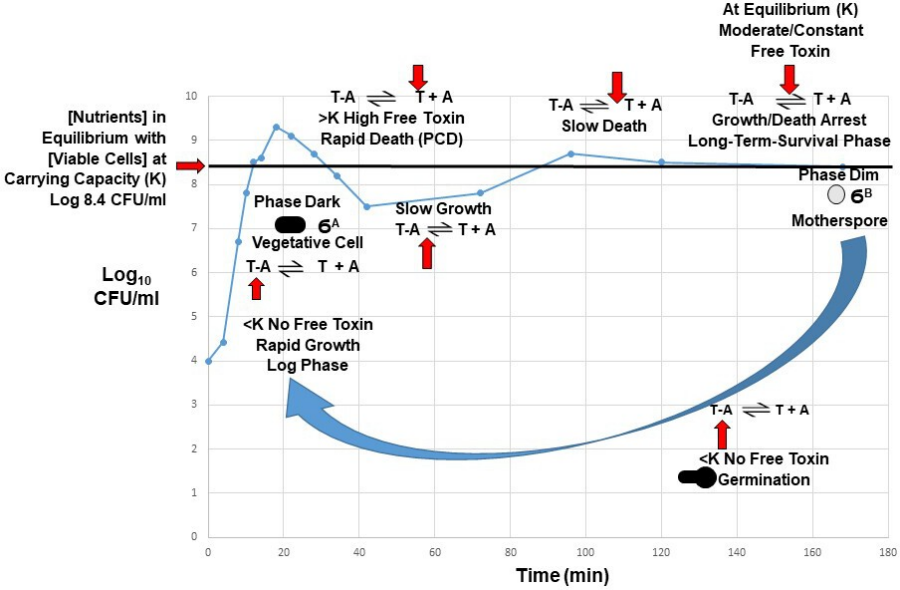
Results of *Listeria monocytogenes* growing in fresh TSBYE at 35°C. Data were generated by inoculating a low concentration of viable cells into fresh TSBYE. Biological replicates of this experiment repeatedly showed the same trend in the blue line. Changing intracellular nutrient concentrations continually shift the position of the T − A ↔ T + A equilibrium throughout the life cycle (vertical red arrows), which results in different intracellular levels of antitoxin and thus free toxin, which produces a damped oscillation of growth, death, growth, growth/death arrest (figure 1). This enables the population to maximize fitness by reproducing at a maximum rate in the log phase when nutrients are in excess and preserving maximum nutrients by dying rapidly when nutrients become limiting above the carrying capacity (*K*) and then rapidly reaching *K* in the form of dormant and stress-tolerant motherspores (figure 3). The large blue arrow indicates germination of motherspores after nutrients have been replenished. Differentiation into vegetative cells is controlled by the primary sigma factor ϬA, and differentiation into stress-tolerant motherspores is controlled by ϬB, the alternative general stress response sigma factor in Gram-positive bacteria. Cultures were plated on tryptic soy agar containing 0.6% yeast extract (TSAYE) and incubated at 35°C for 48 h prior to counting colonies. See figure 5 for the integrated NRCS model, which shows how TASs and sigma factors may work in concert to efficiently regulate the above emergent properties of growth, death, growth/death arrest and germination throughout the life cycle of free-living bacteria.

**Figure 3.**
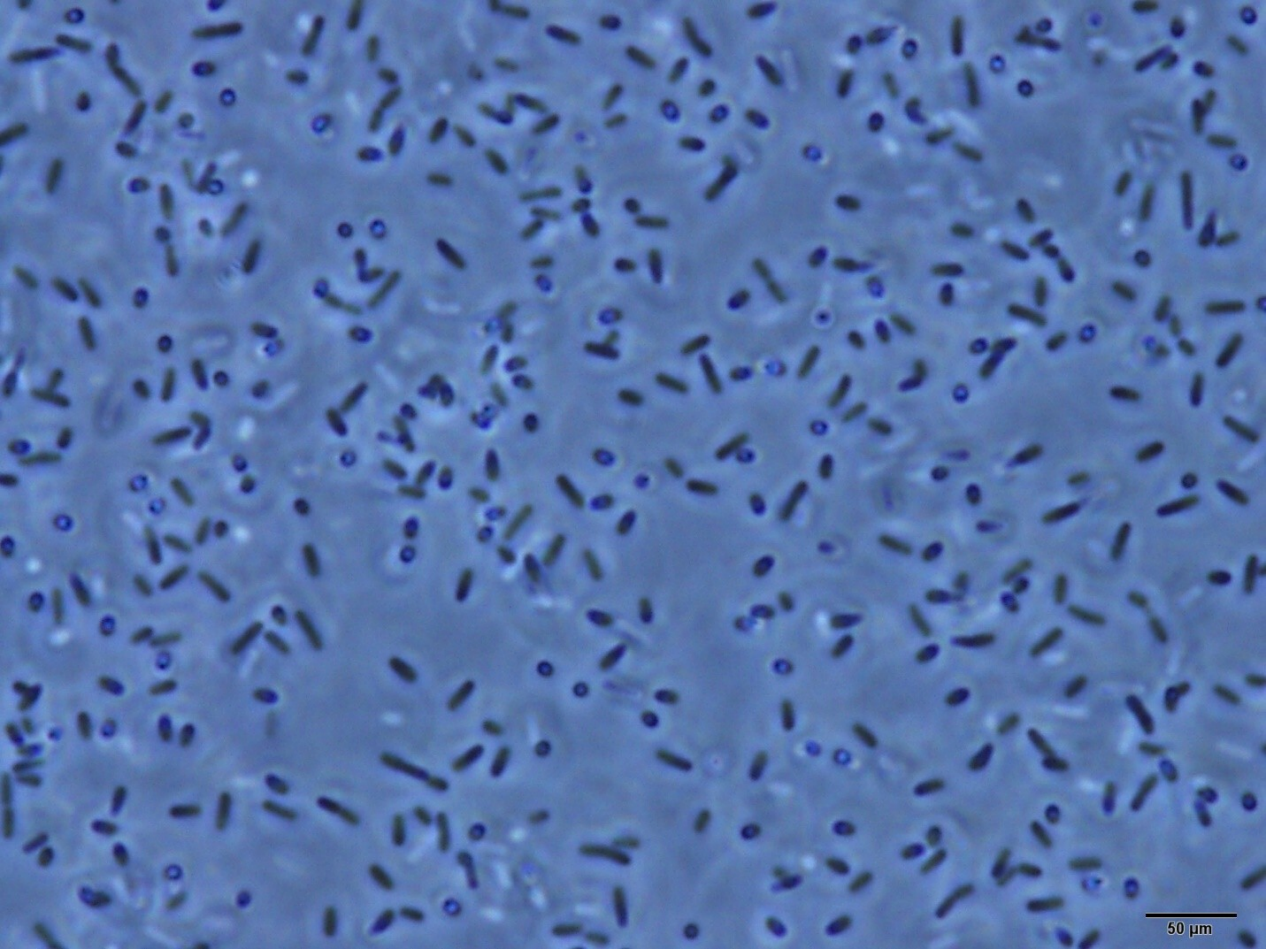
A 1000× phase-contrast microscopy image of the ‘non-spore forming’ bacterium *L. monocytogenes* grown in TSBYE for 4 days at 37°C, showing rod-shaped, phase-dark vegetative cells and coccoid-shaped, phase-dim motherspores. Dull white cells are those that are not in focus.

**Figure 4.**
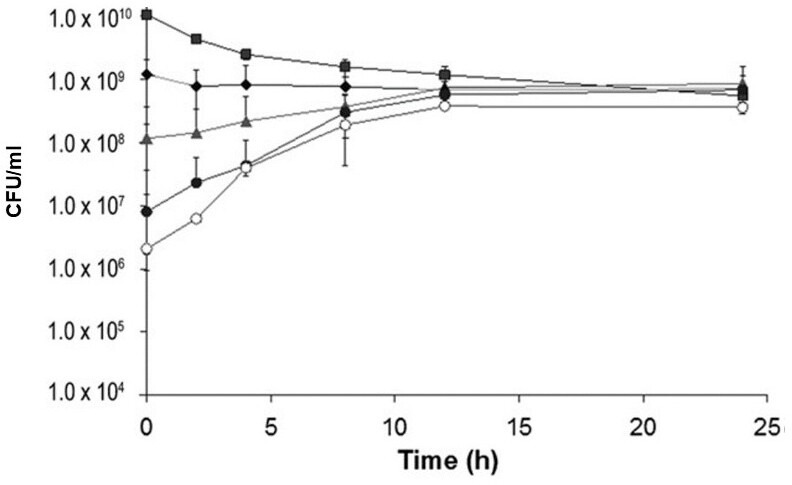
Effect of initial viable cell density on the growth (initial viable cell density <*K*) or death (initial viable cell density >*K*) of *L. monocytogenes* in spent TSBYE incubated at 35°C. Cultures were plated on tryptic soy agar containing 0.6% yeast extract (TSAYE) and incubated at 35°C for 48 h prior to counting colonies. Data were generated by adding different concentrations of viable cells to spent TSBYE (from Wen *et al*. [161] with permission).

**Figure 5.**
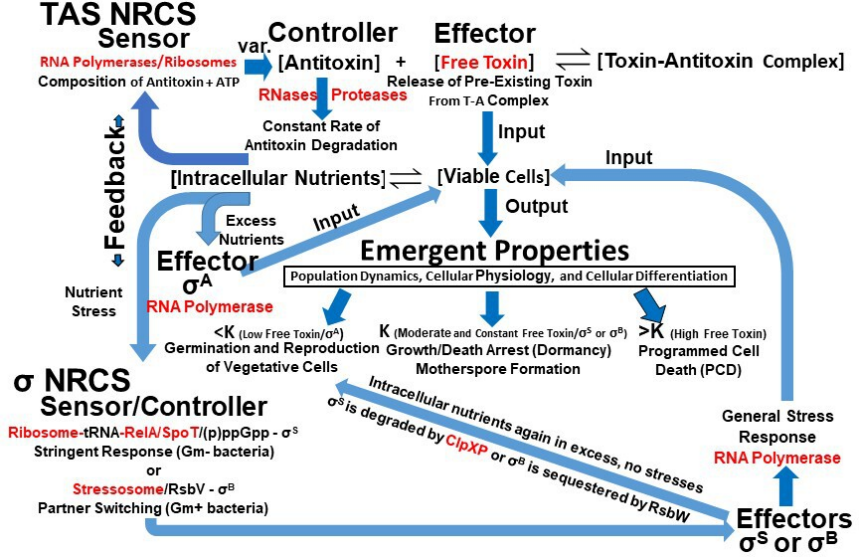
Integrated NRCS model showing how chromosomal TASs and sigma factors may work in concert to optimize the fitness of free-living bacteria. Intracellular nutrient concentration feeds back to sensors, controllers and effectors, which regulate the emergent properties of population dynamics, cellular physiology and cellular differentiation throughout the life cycle. Enzymes that catalyse the integrated NRCS are shown in red. Top, TAS NRCS: Changing intracellular nutrient concentration is sensed by RNA polymerases or ribosomes in conjunction with antitoxin composition and ATP, resulting in varying (var.) concentrations of antitoxin and thus free toxin. Bottom, Sigma Factor NRCS: When nutrients are in excess, σA directs RNA polymerases (RNAPs) to transcribe genes responsible for the reproduction of vegetative cells. Nutrient stress is sensed by the ribosome tRNA–RelA complex (Gram-negative bacteria) or the stressosome (Gram-positive bacteria), which activate alternative sigma factors σS or σB, respectively, which subsequently redirect RNAPs to transcribe genes of the general stress response, which work in concert with TASs to form dormant and stress-tolerant motherspores at *K* (figure 2). When intracellular nutrients are again in excess and there are no stresses, σS is degraded by ClpXP or σB is sequestered by RsbW, thus activating σA, resulting in germination and growth.

One specific major unresolved debate is whether the chromosomally encoded TA toxin MazF causes PCD [31,55,98] or growth arrest [4,65,99]. Amitai *et al*. [100], Lah *et al*. [101] and Yamaguchi & Inouye [102] all concluded that because MazE is a labile protein that is degraded by the cellular protease ClpAP [31], prevention of MazF-mediated cell death requires the continuous production of the antitoxin MazE, and, therefore, stressful conditions that prevent the expression of *mazEF* should trigger cell death [7,44,100]. In support of this, Engelberg-Kulka’s group demonstrated that many different stressful conditions cause MazF-mediated cell death including: (i) extreme amino acid starvation leading to the production of ppGpp [31,98]; (ii) inhibition of transcription and/or translation by antibiotics such as rifampin, chloramphenicol and spectinomycin under specific growth conditions [103], which was confirmed by Kwan *et al*. [104]; (iii) inhibition of translation by the Doc protein of prophage P1 [105]; (iv) DNA damage caused by thymine starvation [103] as well as by mitomycin C, nalidixic acid and UV irradiation [42]; and (v) oxidative stress (H_2_O_2_) [42]. Numerous other authors reported that they could not repeat Engelberg-Kulka group’s finding that ectopic overexpression of MazF caused PCD [100] or they argued that loss of the ability to form colonies upon *mazF* overexpression was due to growth arrest or the formation of viable but non-culturable cells [4,50,56,95,96]. Therefore, these latter authors maintained that the main biological function of MazF and other TA toxins is in growth arrest and persister cell formation, not PCD [50,75]. The Engelberg-Kulka group countered that the above-mentioned authors may not have observed MazF-mediated cell death because they failed to ectopically overexpress MazF toxin long enough to allow cells to reach a ‘point of no return’ [100]. Numerous authors have since confirmed that many chromosomal TASs act as bona fide toxin–antitoxins by demonstrating that ectopic overexpression of toxin causes growth arrest and/or cell death and co-expression of their cognate antitoxin neutralizes the effect of the toxin [14,95,106,107]. However, it is important to keep in mind that ectopic overexpression of TA toxins is an artificial system [5], which probably does not accurately reflect what is occurring in wild-type cells under normal physiological conditions [15,108]. On the contrary, as will be shown later in our TAS-regulated NRCS model, it is the rate of new antitoxin synthesis, not the rate of new toxin synthesis, that controls the concentration of free toxin under normal physiological conditions, which subsequently determines the fate of wild-type free-living bacteria throughout the life cycle.

Pandey & Gerdes [109] argued that it is counterintuitive that single-celled organisms should encode a plethora of suicide genes (TA toxins) [109]. However, William Hamilton developed inclusive fitness theory and derived Hamilton’s rule (*rB* > *C*), which states that altruistic traits like PCD will evolve when relatedness (*r*) between kin multiplied by benefit (*B*) to the beneficiary is greater than the cost (*C*) to the altruist [110]. The equation implies that the more closely related organisms are, the more likely it is that altruistic traits will evolve. Since bacteria reproduce by binary fission and thus are highly clonal, the relatedness within populations of bacteria is very close to 1, which reduces Hamilton’s rule for bacteria to *B* > *C*, thus increasing the likelihood of PCD evolving in bacteria. TA toxins in the chromosomes of free-living bacteria are thought to cause PCD because the death of altruistic cells would enable the remaining viable cells to gain access to nutrients critical for survival and reproduction and thus enhance biological fitness [111]. The long-standing and highly polarized debate over whether TA toxins cause PCD *or* growth arrest may have prevented many scientists in the field from considering that they may actually cause both of these phenomena in order to preserve nutrients required for long-term survival [112]. Therefore, Hamilton’s inclusive fitness theory supports chromosomally encoded TA toxins being altruistic agents that enhance the fitness of bacteria by causing PCD. The phenomena of PCD and cellular differentiation in free-living bacteria caused some scientists to view them as multicellular organisms [55,93,113]. However, the long-standing discussions about whether and why TASs in the chromosomes of wild-type bacteria might cause PCD under normal physiological conditions and whether free-living bacteria really are multicellular organisms remain highly debated and unresolved controversies.

## 4. The principle of population and *r*/*K* selection theory

Explaining the stability and persistence of populations remains one of the most difficult challenges confronting twentieth-century ecologists [38]. In 1798, Thomas Robert Malthus published his famous essay entitled ‘The Principle of Population’, in which he argued that biological populations increase geometrically, while resources (food production) increase arithmetically [114]. Based on this premise, Malthus concluded that biological populations soon approach or exceed a point (Malthusian catastrophe/trap/crisis) where nutrients become limiting and populations must eventually decrease to ‘the level of the means of subsistence’, which is now termed carrying capacity (*K*), which is defined as the maximum population an environment can support or the upper asymptote of the sigmoid curve described by the logistic equation (equation (4.1)) [115]. Malthus realized that as biological populations approach or exceed *K*, they quickly use up food resources critical for sustaining the population, and thus populations would rapidly reach *K* by either preventive or positive checks [114]. Preventive checks can potentially prevent biological populations from exceeding *K*; however, due to what Malthus called ‘the superior geometric power of reproduction’, populations typically exceed *K*, and then positive checks reduce the population to *K* [114]. Pierre Verhulst, a Belgian mathematician, was inspired by Malthus’s essay to derive the logistic equation (equation (4.1)), which describes population growth rate at any time (*t*) as a function of current population size (*N*), intrinsic rate of natural increase (*r*) and carrying capacity (*K*) [116]. Hutchinson [117] later realized that rapidly growing biological populations cannot act instantaneously and thus would first overshoot *K* and then undergo a damped oscillation before reaching *K* [117]. To accommodate this type of delay-driven damped oscillation, Hutchinson incorporated a time lag term (*τ*) in the logistic equation to create a delay logistic equation (equation (4.2); [114]). However, it is important to point out that in both logistic equations given below, biological populations were assumed to be regulated by ‘external’ checks, not by ‘internal’ regulatory systems encoded within an organism’s genome [113,114,116,117].


(4.1) Logistic equation: dN/dt=rN(1−N/K).

(4.2) Delay logistic equation: dN/dt=rN[1−N(t−τ)/K].

Malthus’ essay ‘The Principle of Population’ also provided both Charles Darwin and Alfred Russel Wallace with the mechanism (competition for nutrients and struggle for survival) they needed to derive their now famous theory of evolution by means of natural selection, which is based on biological fitness. Biological fitness is defined as reproductive success or the ability to transfer genetic information to the next fertile generation, compared with other organisms in the same environment. Put simply, this theory states that those organisms that possess the greatest fitness (ability to survive and reproduce) will evolve into the future, while those organisms that are less fit will be out-competed and eventually become extinct [118]. It is important to keep in mind that for bacteria, *reproduction* as vegetative cells in the log phase and *delayed reproduction* as dormant and stress-tolerant cells in the long-term-survival (LTS) phase both contribute to overall reproductive success and thus fitness.

In the 1960s, MacArthur and Wilson used the *r* and *K* concepts in the logistic equation to develop *r*/*K* selection theory to explain how organisms evolve life history strategies to become either *r*-selected or *K*-selected [119]. In that theory, *r*-selected species evolve in environments where nutrients are in excess and competition is low, while *K*-selected species evolve in environments where there is intense competition for limited nutrients. *r*/*K* selection theory was further expanded by Pianka, who argued that no organism could be perfectly *r*- or *K*-selected, but organisms must compromise and occupy niches along an *r*–*K* continuum [120]. Similarly, Brommer [121] argued that trade-offs exist between reproduction (*r*) and survival (*K*), such that organisms must reduce their reproductive success to enhance their survival [121]. However, most authors in the *r*/*K* and life history fields only applied *r*/*K* selection and life history trade-off theories to sexual organisms like plants and animals but did not apply them to asexual organisms like bacteria, which can rapidly and efficiently shift their gene expression profiles to achieve temporally distinct *r* and *K* lifestyles, thereby avoiding a trade-off between reproduction and survival and thus optimizing both *r* and *K* and biological fitness.

## 5. Dogma I: spore-forming versus non-spore-forming bacteria

### 5.1. Spores and the bacterial life cycle

Sporulation is common in organisms throughout the tree of life, from bacteria and protozoa to plants and fungi, and facilitates their dispersal and/or survival against various stresses [122]. A few genera of free-living bacteria produce metabolically inert and extremely stress-tolerant endospores following exhaustion of nutrients [123], which allows them to survive in nature for hundreds to thousands or even millions of years [124]. Reder *et al*. [125] and Ayala *et al*. [126] both reported that endospore formation in *Bacillus* is inhibited by the alternative sigma factor Ϭ^B^ [125,126], which directs transcription instead towards genes associated with the general stress response, which is induced by both physical and nutrient stress [127,128]. Reder *et al*. [125] concluded that endospore formation is a costly ‘last resort’ adaptive response to nutrient starvation, while the Ϭ^B^-regulated general stress response is the preferred response to physical stress during long-term survival [122,129]. Both endospore formation and the general stress response are triggered by nutrient starvation and provide tolerance to numerous other types of stress, such as heat, freezing, dehydration, high and low pH, osmotic stress, UV light, toxic chemicals, high pressure and antibiotics [123,125]. Therefore, both endospore formation [123] and the general stress response [127,128,130] can be viewed as ‘bet-hedging’ responses to self-induced nutrient starvation, but they are regulated by different numbers and types of alternative sigma factors.

Ferdinand Cohn and Robert Koch were the first to describe the life cycle of free-living bacteria when studying endospore-forming bacteria in the genus *Bacillus* [131]. Koch demonstrated that the soil-borne bacterial pathogen *Bacillus anthracis* cycles between rod-shaped vegetative (reproducing) cells when nutrients are in excess and dormant (non-reproducing) and stress-tolerant endospores when nutrients become limiting. The life cycle of endospore-forming bacteria begins when nutrient stress triggers asymmetric septation and cellular differentiation of vegetative cells into mother cell and daughter cell (forespore). The larger mother cell subsequently engulfs the smaller forespore, and the latter eventually transforms into a coccoid-shaped, dormant and stress-tolerant endospore. Cellular differentiation into vegetative cells and endospores is well known to be under the control of sigma factors, with the primary sigma factor (Ϭ^A^) regulating the transcription of hundreds of genes associated with growth of vegetative cells and alternative sigma factors Ϭ^F^, Ϭ^E^, Ϭ^G^ and Ϭ^K^ regulating the transcription of hundreds of genes associated with differentiation of vegetative cells into dormant and stress-tolerant endospores [123]. Multiple alternative sigma factors are required for endospore formation due to the need for temporally separated gene expression in the mother cell and forespore compartments [132]. Endospores are highly refractile (phase bright), which is thought to be due to the dehydrated nature of the spore core [133]. The life cycle is completed when endospores germinate back into non-refractile (phase dark) stress-sensitive vegetative cells when excess nutrients again become available [134]. The transition between vegetative cells and endospores is accompanied by dramatic changes in (i) gene expression, (ii) cellular physiology, (iii) cellular morphology, and (iv) stress tolerance [123,135]. A few other genera of free-living bacteria, like those in the myxobacteria and actinomyces, also form highly refractile spores, but not endospores, after reductive division and/or shrinkage and condensation of vegetative cells [135,136].

### 5.2. ‘Non-spore forming’ bacteria and the bacterial ‘growth curve’

Unlike the relatively few spore-forming bacteria [136], the majority of free-living bacteria are classified as ‘non-spore forming’ [137]. However, like the formation of endospores, upon nutrient starvation, ‘non-spore forming’ bacteria also differentiate into coccoid-shaped cells that are tolerant to numerous environmental stresses [138–143]. Downregulation of macromolecular synthesis and induction of stationary phase morphogenes cause stationary phase cells to take on their classical coccoid morphology [142]. This explains why *rpoS*^−^ mutants [141] and *relA^−^/spoT*^−^ mutants of *E. coli*, which cannot synthesize (p)ppGpp and thus cannot activate Ϭ^S^ [142], are both unable to form coccoid cells following amino acid starvation. Due to the classification of most bacteria as ‘non-spore forming’, the population dynamics of these free-living bacteria has long been described as comprising the four phases (lag, log, stationary and death) of the classic ‘bacterial growth curve’, a concept that remains firmly embedded in the field of microbiology to this day [144]. However, the bacterial growth curve concept is problematic because growth (reproduction) only occurs in one of the four phases, the log phase. In addition, since death is the last phase of the bacterial growth curve, there is no apparent life cycle; however, paradoxically, these ‘non-spore forming’ bacteria have survived enumerable cycles of feast and famine for billions of years. Due to the above long-held dogmas of ‘non-spore forming’ bacteria and the ‘bacterial growth curve’ inaccurate, confusing and/or nonsensical terms like ‘vegetative dormancy’ [125] and ‘non-differentiating bacteria’ [140] have been used to describe ‘non-spore forming’ free-living bacteria, even though they clearly differentiate into dormant, coccoid-shaped, stress-tolerant cells when nutrients become limiting [139,140,142,143,145,146] and subsequently germinate back to a vegetative (reproducing) state when conditions become favourable again [143,147,148].

Based on the above, alternative sigma factors that induce the general stress response in Gram-negative and Gram-positive bacteria may work in concert with TASs to form dormant, coccoid-shaped and stress-tolerant cells at the carrying capacity (*K*), which has been termed the LTS phase [143]. Similar to endospores, such cells in the LTS phase are more tolerant to various stresses, including heat, high pressure, desiccation, UV light, chlorine and cold plasma [143,149,150]. Lange & Hengge-Aronis [141] observed that shrinkage of *E. coli* from rods to coccoid-shaped cells only occurred at high optical densities, where nutrients would be depleted. Based on the comparison of Ϭ^S−^ mutant and wild-type cells, they concluded this phenomenon must be due to a complex mechanism under the control of Ϭ^S^ [141]. As mentioned previously, the need for temporally distinct gene expression patterns that regulate the complex interplay between mother cell and forespore explains why endospore formation requires four different alternative sigma factors [132]. In contrast, only one alternative sigma factor (Ϭ^S^ or Ϭ^B^) is needed to regulate hundreds of genes associated with the general stress response in Gram-negative bacteria [151] or Gram-positive bacteria [126], respectively. This results in the shrinkage and condensation of rod-shaped cells into dormant, stress-tolerant and coccoid-shaped cells [141,142]. We now term these dormant, coccoid-shaped and stress-tolerant cells in the LTS phase motherspores, as they meet all the criteria of a spore (table 1) but form directly by shrinkage and condensation of mother cells, in contrast to endospores or ‘daughterspores’, which form by engulfment of forespores (daughter cells) by mother cells. Similar to the above proposed role of Ϭ^S^ in motherspore formation in Gram-negative bacteria, Lee *et al*. [152] demonstrated that Ϭ^B^ is required for spore formation in the Gram-positive bacterium *Streptomyces coelicolor* [152]. Figure 3 shows rod-shaped vegetative cells and coccoid-shaped motherspores of the Gram-positive ‘non-spore forming’ bacterium *Listeria monocytogenes* after incubation in tryptic soy broth with 0.6% yeast extract (TSBYE) for 4 days at 35°C. Unlike endospores, which are highly refractile and thus ‘phase bright’ and vegetative cells, which are non-refractile and thus ‘phase dark’, motherspores are slightly refractile or ‘phase dim’ (figure 3). This is like a species of *Streptomyces*, which also transitions from phase-dark vegetative cells into phase-dim spores upon nutrient depletion [136,153]. Unlike metabolically inert endospores but like persister cells [85,154–157], motherspores may possess low levels of metabolic activity and thus require a steady supply of nutrients to remain viable during long-term survival in nature.

**Table 1.** Motherspores meet all the criteria for spores.

**criteria for spores**	motherspores
coccoid shape	yes
dormant (no growth with little or no metabolic activity)	yes
induced to form by nutrient depletion and other stresses	yes
resistant to numerous environmental stresses	yes
formation regulated by alternative sigma factors	yes
cellular differentiation from phase dark vegetative cells to phase-dim to phase-bright spores	yes
germinate back to vegetative cells when conditions for growth become favourable again	yes

## 6. Dogma II: unicellular versus multicellular organisms: programmed cell death and cellular differentiation are multicellular traits

### 6.1. Chromosomally encoded toxin–antitoxins may help bacteria function as multicellular organisms

Another historic, but now questionable, dogma in microbiology is the long-held perception that bacteria are unicellular organisms. However, many authors [55,113,158,159] now argue that bacteria should be considered multicellular organisms because they possess traits associated with multicellularity, such as PCD and cellular differentiation. In free-living bacteria, PCD has been associated with chromosomally encoded TASs [55,89,92], and cellular differentiation has been associated with alternative sigma factors that regulate both spore formation in a few Gram-positive bacteria [123] and the formation of ‘coccoid cells’ in numerous ‘non-spore forming’ bacteria [141,142] when nutrients become limiting.

Some authors have argued that TA toxins like MazF operate *in vivo* as altruistic agents by lysing their host cells, and thus their death ‘provides’ nutrients that enhance the fitness of closely related survivors [31,55,160]. However, both Lioy *et al.* [68] and Penesyan *et al.* [159] demonstrated that at physiological levels, zeta (PezT) toxin caused ‘death from within’ [38], not death by cell lysis. Similarly, Yamaguchi *et al.* [44] and Penesyan *et al.* [159] also reported no cell lysis during the death of *Streptococcus mutans* and *E. coli*, respectively, following overproduction of MazF. Data from our laboratory (S.J.K.) also revealed no evidence of cell lysis (no increase in 260 nm absorbing material and no change in optical density at 600 nm, data not published) and no microscopically visible loss of cell integrity (figure 3), following rapid death of *L. monocytogenes* after reaching a peak viable cell density of approximately log 9.5 CFUs ml^−1^ in TSBYE [161].

Wen *et al*. [161] modelled the maximum specific population growth rate (*r*) of *L*. *monocytogenes* in TSBYE at 35°C using the logistic equation (equation (4.1)) and predicted it to be 0.8 h^−1^, and *K* was predicted to be log 8.6 CFUs ml^−1^ for both spent TSBYE and fresh TSBYE [161]. Data for spent TSBYE (figure 4) closely fitted the logistic equation when the initial viable population density was adjusted below or above *K* [161], while data for fresh TSBYE showed a damped oscillation after the viable population density overshot *K* (figure 2, data not published). The data shown in figures 2 and 4 may have been due to viable cells responding to *intracellular nutrient concentration*, rather than viable population density *per se*, as stated in Wen *et al*. [161]. This may explain why growth and death of *L. monocytogenes* were highly predicted by the logistic equation (equation (4.1)) when initial viable cell density was varied above and below *K* in spent TSBYE (figure 4) [161]. Therefore, in our integrated NRCS model (figure 5), *varying intracellular nutrient concentration* is the *feedback* that determines antitoxin concentration, which determines the concentration of free toxin, which ultimately determines cell fate throughout the life cycle of free-living bacteria (figure 2). In the passive translation-responsive model Ramisetty [52] tied nutrient resources to dynamic TA regulation; it did not include the concept of cybernetics, including feedback (changing intracellular nutrient concentration), ultimately regulating population dynamics and cellular physiology throughout the life cycle [52] (figures 2 and 5). In addition, the passive translation-responsive model was based on type II TASs and thus did not apply to those TASs based on RNA antitoxins. In contrast to the translation-responsive model, the NRCS model put forward in this paper may explain the function of all TASs, both those based on transcription (synthesis of RNA antitoxins) and those based on translation (synthesis of protein antitoxins), as the rates of both transcription and translation respond to intracellular nutrient concentration [32,162].

## 7. The nutrient-responsive cybernetic system model

### 7.1. Toxin–antitoxin systems may function as nutrient-responsive cybernetic systems that efficiently regulate population dynamics and cellular physiology throughout the life cycle

Although the characteristics of toxins and antitoxins have been well-studied, their primary biological function has remained elusive. This may be due to toxins and antitoxins possessing previously unrecognized emergent properties, which are defined as properties that do not belong to individual components themselves but only become apparent when viewed as interacting components within a larger system [163]. Systems that possess emergent properties are sometimes referred to as complex adaptive systems, which are defined as systems made up of individual agents that can act in unpredictable ways, and whose actions are connected in such a way that one agent’s actions can change the context for other agents [164]. Within the broad field of complex adaptive systems, cybernetics is a cross-disciplinary field that focuses on control in machines and biological organisms [165]. The core concept of cybernetics is circular causality or feedback, where the observed outcomes of actions are taken as inputs for further action in ways that support the pursuit and maintenance of optimal conditions [165–167]. For example, in our NRCS model, intracellular nutrient concentration feeds back into the system and controls the intracellular concentration of antitoxin and thus concentration of free ‘active’ TA toxins, which regulate various emergent properties of free-living bacteria throughout the life cycle (figures 2 and 5). If high levels of intracellular T–A complexes exist following the log phase, PCD at cell densities above *K* (where intracellular nutrients are limiting) may be due to both the lack of antitoxin synthesis and a constant rate of antitoxin degradation, which results in the release of high levels of free toxins from pre-existing T–A complexes (figures 1 and 2; [13,18]). As mentioned above, some researchers [22,23,88] have argued that free TA toxins are not released under normal physiological conditions when antitoxins are bound tightly to toxins in a folded state and therefore are protected from degradation. In our NRCS model at viable cell densities above *K*, the equilibrium dramatically shifts due to a large decrease in intracellular nutrient concentration and thus antitoxin concentration, which results in the dissociation (release) of high concentrations of free toxins and subsequent PCD [18,168] (figures 1, 2 and 4). The damped oscillation of growth, death, growth/death arrest observed in fresh TSBYE (figure 2) may be due to both time needed for the degradation of existing antitoxin and release of free toxin above *K* and time needed for the synthesis of new antitoxin and sequestering of toxin below *K*, which explains why the damped oscillation data in figure 2 are predicted by the delay logistic equation (equation (4.2); [117]).

RNA polymerases and ribosomes, along with antitoxin composition, may serve as biological ‘sensors/controllers’ that both ‘sense’ the availability of intracellular nutrients critical for antitoxin synthesis and then ‘control’ the concentration of antitoxin and thus free TA toxin, which subsequently determines whether growth, PCD, growth/death arrest or germination occurs (figures 2 and 5). Interestingly, while protein antitoxins are small, they are composed of 18−20 of the 20 known amino acids. For example, MazE in the Gram-negative bacterium *E. coli* is only missing cysteine and tyrosine, which are infrequent in *E. coli* proteins [169] but can be synthesized by *E. coli*. Likewise, *L. monocytogenes*, *B. subtilis* and *S. aureus* contain MazE molecules that are only missing the amino acid tryptophan, which is the least frequent amino acid in bacterial proteins [169] but can be synthesized by these Gram-positive bacteria. Therefore, the intracellular concentration of amino acids and ATP may determine the rate of MazE synthesis, which controls the intracellular concentration of MazE and thus the concentration of free MazF, which controls cell fate. A constant rate of antitoxin degradation and variable rates of antitoxin synthesis would allow the concentration of antitoxin and thus the concentration of free TA toxin to be controlled in a continuous analogue manner [52]. This is consistent with the smooth kinetics of growth and death observed as viable cells of *L. monocytogenes* approach growth/death arrest (dormancy) at *K* (figures 2 and 4). While this paper focuses mainly on the type II TAS MazEF, the proposed nutrient-responsive model may also apply to all TASs, both those that use protein antitoxins and those that use RNA antitoxins. Protein antitoxins are composed of five of the six essential elements for life (C, H, N, O and S), and ATP, which is required for protein synthesis, contains the remaining element P, while RNA antitoxins are composed of the following five essential elements for life: C, H, N, O and P. Therefore, RNA polymerases and ribosomes that control the synthesis of RNA and protein antitoxins, respectively, may serve as ‘sensors’ of essential nutrients, which control the concentration of antitoxins and thus free toxins, which subsequently serve as effectors of cell fate (figures 2 and 5). Such a TAS-regulated *NRCS* would include three *components* and their corresponding *enzymes* that rapidly and efficiently regulate population dynamics and cellular physiology throughout the life cycle: (i) a ‘sensor’ (*RNA polymerases or ribosomes* in conjunction with antitoxin composition and ATP); (ii) a ‘controller’ (antitoxin concentration), which is regulated by the concentration of intracellular nutrients and *RNases/proteases* that constantly degrade antitoxins; (iii) an ‘effector’ (*free TA toxins*), which have pleiotropic effects on various cellular targets depending on their intracellular concentration at various stages in the life cycle (figures 2 and 5). In our NRCS model, free ‘active’ toxins determine the concentration of viable cells, and since only viable cells can synthesize ATP and transport and/or synthesize amino acids and RNA nucleotides, they ultimately determine the concentration of these critical nutrients inside viable cells, which subsequently feeds back to the RNA polymerase or ribosome ‘sensor’ (figure 5). Such an emergent TAS-regulated NRCS may optimize the fitness of free-living bacteria by enabling them to efficiently regulate growth, PCD, growth/death arrest and germination throughout the life cycle, depending on the concentration of essential intracellular nutrients (figures 1, 2 and 5).

### 7.2. Sigma factors may act as nutrient-responsive cybernetic systems to efficiently regulate cellular differentiation throughout the life cycle

While chromosomal TASs may regulate population dynamics and cellular physiology, sigma factors are known to regulate cellular differentiation during reproduction and survival modes of existence. Cellular differentiation following nutrient stress is mediated by alternative sigma factors in both Gram-positive bacteria [123] and Gram-negative bacteria [141,142]. The latter is accomplished by a process known as the stringent response, which lies at the core of the bacterial stress response system [170–172]. During logarithmic growth, the presence of charged tRNAs (tRNAs containing their cognate amino acid) in the A-site of the ribosome stimulates the expression of the primary sigma factor (Ϭ^A^), which directs RNA polymerases (RNAPs) to transcribe those genes responsible for reproduction. Upon starvation, uncharged tRNAs enter the A-site of the ribosome, resulting in tRNA stalling and the formation of a stable tRNA–RelA–ribosome complex, which subsequently activates the enzyme RelA to synthesize the alarmone (p)ppGpp [173]. (p)ppGpp is also synthesized by the enzyme SpoT during carbon, fatty acid, phosphate and/or iron starvation [171]. (p)ppGpp has been shown to subsequently activate the alternative sigma factor Ϭ^S^, which binds to RNAPs and redirects them to transcribe genes of the general stress response [130,151,174]. Directly or indirectly, Ϭ^S^ regulates 10% of the *E. coli* genome (approx. 500 genes) [46]. In Gram-positive bacteria, nutrient and environmental stresses are detected by a stressosome [128] and result in a process called partner switching [175], which leads to the release of the alternative sigma factor Ϭ^B^, which subsequently redirects RNAPs to transcribe more than 150 genes of the general stress response [126,149,176–180]. Like endospore formation, the general stress response is a global response to nutrient and environmental stresses, which subsequently provides both Gram-negative bacteria [181] and Gram-positive bacteria [182] with protection against many different types of stress.

Similar to antitoxins, anti-sigma factors are also co-transcribed with their cognate sigma factors [179,183,184], and this pairing creates a negative feedback loop, maintaining proper levels of both factors, as there can only be one anti-sigma factor per sigma factor that is transcribed [185]. Nutrient-responsive negative feedback controls the type of sigma factor that predominates during reproduction or survival modes of existence [126,180,186]. When intracellular nutrients are limiting, the primary anti-sigma factor binds onto its cognate sigma factor (Ϭ^A^) and keeps the latter inactive, while free alternative sigma factors (Ϭ^S^ and Ϭ^B^) direct transcription of those genes responsible for the general stress response [130,174,177,187], which may result in the formation of dormant, stress-tolerant, coccoid-shaped motherspores (figures 2, 3 and 5).

In addition to turning on Ϭ^S^ or Ϭ^B^ following nutrient stress, these alternative sigma factors are also rapidly inactivated via partner switching when excess nutrients again become available. In Gram-positive bacteria, RsbW shifts from binding onto RsbV to binding onto and sequestering (inactivating) Ϭ^B^, resulting in the predominance of Ϭ^A^ and reproduction (figure 5; [126,175,180]). Likewise, in Gram-negative bacteria, RssB switches from binding to IraP to binding to Ϭ^S^, which results in the degradation (inactivation) of Ϭ^S^ by ClpXP, leading to the predominance of Ϭ^A^ and reproduction (figure 5; [186]). Fast, proteolytic degradation and slower, broad transcriptomic changes regulated by alternative sigma factors give cells the mechanistic repertoire to dynamically adjust the bacterial proteome to provide tolerance to various stresses [188]. Gram-positive bacteria [180] and Gram-negative bacteria [186] use negative feedback based on nutrient availability to decide whether primary or alternative general stress response sigma factors are active, and thus, together with TASs, might function as an *Integrated NRCS* (figure 5) that enables free-living bacteria to rapidly cycle between stress-sensitive vegetative cells and stress-tolerant motherspores (figure 2). Both *rpoS*^−^ mutants of *E. coli* [141] *relA^−^/spoT*^−^ mutants of *E. coli* [142] were unable to synthesize Ϭ^S^ and therefore could not transition from rod-shaped cells in the log phase to coccoid-shaped cells in the stationary phase. Similarly, *sigB*^−^ mutants of the Gram-positive bacterium *S. coelicolor* were unable to form spores upon nutrient stress [189]. The above-mentioned mutation results are consistent with the general stress response sigma factors Ϭ^S^ and Ϭ^B^ regulating spore formation upon nutrient stress (figures 2 and 5). If true, all free-living bacteria may form spores, just different kinds of spores [136], and thus all possess a life cycle that enables their long-term survival in nature.

## 8. Putting it all together

While much is known about the molecular mechanisms of TASs, little is known for sure about their main biological function. This may be due to toxins and antitoxins typically being studied in isolation, rather than as part of a large interacting whole system that includes molecular, cellular and population (multicellular organism) levels [190–192]. Berkvens *et al*. [193] recently took a systems biology approach to modelling persister cell formation [193]. They emphasized the need for an overarching framework based on molecular, physiological and evolutionary perspectives related to the fitness merits of persister cell formation. They logically argued that TASs, sigma factors and (p)ppGpp interact to regulate stress control in free-living bacteria. Their underlying premise was that free-living bacteria switch between two distinct fitness strategies: reproduction via vegetative cells and survival via persister cells. However, by focusing on the concept of persister cells, they failed to consider the fundamental biological concepts of spore formation and life cycle. Berkvens *et al*. [193] concluded that the concentration of free toxin is determined by toxin expression and the induction of cellular proteases [193]. In contrast, in our NRCS model, the varying intracellular nutrient concentration determines the intracellular antitoxin concentration, which determines the intracellular concentration of free toxin, which determines cell fate throughout the life cycle (figures 2 and 5). In our model, the concentration of free toxins is low below *K* because cells have ample intracellular nutrients to express both toxins and antitoxins; however, antitoxins are expressed at a slightly higher rate to keep the toxin neutralized and thus permit growth. In contrast, above *K*, the concentration of free toxins dramatically increases because cells lack the intracellular nutrients needed to express both toxins and antitoxins, which results in antitoxin degradation, the release of free toxins and death. Some authors in the field have argued that free toxins are not released because antitoxins are not degraded when they are tightly bound to their cognate toxins. In our model, the concentration of free toxins is low below *K* because cells have ample intracellular nutrients to express both toxins and antitoxins; however, antitoxins are expressed at a slightly higher rate than toxins to keep the toxin neutralized and thus permit growth. In contrast, above *K*, the concentration of free toxins dramatically increases because cells lack the intracellular nutrients needed to express both toxins and antitoxins, which results in antitoxin degradation, the release of free toxins and death. Therefore, the increase in free toxin during stress is not due to a difference in toxin and antitoxin *expression* but is due to a *LACK of toxin and antitoxin expression, which together with the degradation of free antitoxin leads to the dissociation of antitoxin from the T–A complex and the subsequent release of free toxin*. Therefore, the integrated NRCS model directly ties cell fate back to intracellular nutrient concentration, which enables free-living bacteria to rapidly and efficiently convert nutrients into reproductive propagules throughout the life cycle and thus optimize biological fitness. The continued focus on variable rates of antitoxin degradation, ectopic overexpression of TA toxins and deletion of chromosomal TASs to determine their biological function has prevented many scientists in the field from considering mechanisms that control the activity/concentration of free toxin via the *rate of antitoxin transcription or translation* in *wild-type cells* under *normal physiological conditions*. This is especially important at *high viable cell densities above K*, where intracellular nutrient concentration and thus antitoxin concentration would be expected to dramatically decrease, and therefore the concentration of free toxin to dramatically increase (figure 2). For example, Tashiro *et al*. [194] demonstrated that dormancy and persister cell formation in *E. coli* due to the TA toxin RelE were enhanced at high viable cell density [194]. Density-dependent factors impinge more severely upon populations when their density is high and must provide mechanisms by which they return to their equilibrium density following a perturbation [38]. In our integrated NRCS model, chromosomal TASs and sigma factors represent two tightly interconnected NRCSs that enable free-living bacteria to reproduce rapidly as vegetative cells when nutrients are in excess, but then rapidly and efficiently transition to dormant and stress-tolerant motherspores when nutrients become limiting (figures 2 and 5). Therefore, rather than identifying two different ‘types’ of persister cells whose formation was proposed to be either ‘spontaneous’ or ‘triggered’ [79] in the integrated NRCS model, TASs and sigma factors regulate the population dynamics, cellular physiology and cellular differentiation of two fundamental ‘types’ of free-living bacterial cells throughout the life cycle, namely *vegetative cells* and *spores* (figure 2). The transition between vegetative cells and spores may be a universal and unifying concept in microbiology and explain how all free-living bacteria optimize biological fitness as they cycle between short-term feast and long-term famine.

The integrated NRCS model is consistent with all of the following: (i) the theories of natural selection [118] and inclusive fitness [110]; (ii) the concept of carrying capacity [114]; (iii) the concepts of spore and life cycle [195]; (iv) both TASs and sigma factors primarily responding to nutrient status throughout the life cycle of free-living bacteria [50,172,196,197]; (v) the concepts of emergence [162] and complex adaptive systems [164]; (vi) the concept of cybernetics [165]; (vii) all known physical and chemical properties and molecular functions of toxins and antitoxins [14] and sigma factors and anti-sigma factors [184]; (viii) TA toxins being released following any phenomenon that prevents antitoxin synthesis [4]; (ix) the role of TASs in shutting down various types of metabolism resulting in dormancy [78]; (x) pleiotropic effects (death and growth arrest) occurring at different concentrations of free TA toxins [71,72,102]; (xi) TA toxins enhancing fitness via both PCD [55] and growth arrest [4]; (xii) the role of alternative sigma factors in regulating the differentiation of vegetative cells into stress-tolerant spores [123,189]; (xiii) the multicellular nature of bacteria [113]; and (xiv) the recently proposed passive translation-responsive model [52]. The integrated NRCS model is also consistent with the ubiquitous presence of TASs [4] and *relA/spoT* homologue genes [198] in the chromosomes of all free-living bacteria and their absence in the chromosomes of endosymbiotic bacteria [109,198], as the latter live in nutrient-constant environments and thus have no life cycle and therefore no need for these chromosomally encoded systems.

The TAS-regulated NRCS model is also consistent with the results of Williams & Hergenrother [199], who demonstrated that *in vitro* MazF activity from lysed log phase cells of *S. aureus* constantly increased with time after cell lysis [199]. The *in vitro* results shown in [199, fig. 5] are consistent with the same proposed mechanism that controls viable cell density above *K* (figures 1 and 2). In both cases, the increased release of free MazF toxin from the pre-existing MazE–MazF complex is due to both the lack of new MazE synthesis and the constant degradation of pre-existing MazE antitoxin by cellular proteases. By synthesizing high levels of toxins and even higher levels of antitoxins in the log phase, free-living bacteria would be primed with high intracellular concentrations of neutralized toxin–antitoxin complexes, which upon binding to T–A promoters would inhibit unnecessary further synthesis of toxins and antitoxins, but would be poised to subsequently release high intracellular levels of free toxins when the viable population density exceeds *K*. This results in PCD, which quickly brings the population back down to equilibrium (*K*), where the surviving cells become growth/death arrested due to a constant and moderate concentration of free toxin (figures 2 and 4). Therefore, consistent with the above-mentioned publications of the Engelberg-Kulka group, the NRCS model predicts that factors that inhibit the synthesis of antitoxins will unleash high levels of free TA toxins, resulting in PCD. Strategies that take advantage of this ‘Achilles heel’ of free-living bacteria may result in the development of novel drug therapies that can prevent serious infections caused by a wide variety of bacterial pathogens.

All three components of the TAS-driven NRCS (sensor, controller and effector) contain enzymes that rapidly and efficiently accelerate the attainment of *K* (figures 2 and 4), which maximizes the preservation of essential nutrients. TA toxin-mediated PCD preserves nutrients by rapidly killing cells that have overshot *K*, while TA toxin-mediated growth/death arrest preserves nutrients by rapidly and dramatically reducing the metabolic activity of surviving motherspores at *K* (figure 2). We now term this TAS-regulated NRCS fitness strategy ‘K sensing and control’ (KSC; figures 2 and 4), which represents an evolved, highly efficient auto-regulatory mechanism of population control [200]. At the same time, primary and alternative sigma factors direct enzymes (RNAPs) to catalyse the transcription of genes responsible for cellular differentiation into vegetative cells and spores, respectively [123,179,189] (figure 2). Therefore, the chromosomally encoded, enzymatically catalysed, integrated NRCS model (figure 5) allows free-living bacteria to reproduce as fast as possible as vegetative cells in the log phase and then escape the Malthusian catastrophe when nutrients become limited by rapidly reaching *K* and surviving as dormant and stress-tolerant motherspores (figures 2 and 3), which together enables them to optimize biological fitness.

Finally, the integrated NRCS model resolves many of the long-standing debates, controversies and contradictions concerning the main biological function of chromosomal TASs. It also clarifies many previously proposed hypotheses and integrates them into one coherent unified model that is consistent with the known physical and chemical properties and molecular functions of TASs and sigma factors. Three phenotypes frequently associated with TASs are PSK, PCD and Abi, all three of which result in cell death. In the present model, chromosomally encoded TASs mediate KSC, which includes cell death when populations exceed *K*, but also growth/death arrest (dormancy) when populations reach *K*. While the main biological function of chromosomal TASs may be to optimize the fitness of free-living bacteria throughout the life cycle, this does not preclude the possibility that other related functions of chromosomal TASs may have also evolved, including roles in stabilization of genomic elements, plasmid maintenance, phage inhibition, biofilm formation and virulence/pathogenesis [22,28,39]. For example, PSK and Abi may have evolved when plasmids and cells co-opted the PCD function of KSC for the purposes of plasmid maintenance and phage defence, respectively. The proposed integrated NRCS model (figure 5) is relatively simplistic and lacks many other complex regulatory elements and interactions [47,130,174,180,182,186,201]. For example, Handler & Kirkpatrick recently argued that RpoS-mediated general stress response activation is not a binary gene expression switch, but is able to mediate phenotypic heterogeneity in the stressed population, which increases fitness in that subpopulation [202]. However, even though the integrated NRCS model presented in this paper is relatively simplistic, it provides a novel, rational, coherent model for generating testable hypotheses concerning the biological function of chromosomal TASs and how they may function in concert with sigma factors to enable free-living bacteria to efficiently cycle between reproduction and long-term survival modes of existence. The proposed model would be limited if it were not applicable to all chromosomal TASs and all free-living bacteria that contain chromosomal TASs. The model assumes a continuously shifting equilibrium between T–A complexes and free TA toxins throughout the life cycle, due to the different stabilities of TA toxins and antitoxins and a constant rate of antitoxin degradation. The model is also based on the assumption that intracellular nutrients feed back within the integrated NRCS to control population dynamics, cellular physiology and cellular differentiation throughout the life cycle. If some or all of the above-mentioned assumptions are incorrect, then the proposed integrated NRCS model would be limited or invalid.

## 9. Testing predictions of the toxin–antitoxin system-regulated nutrient-responsive cybernetic system model

The following predictions are based on the response of wild-type free-living bacterial cells to the concentration of critical intracellular nutrients. In general, the model can be tested by varying the intracellular nutrient concentration and then measuring the concentration of antitoxin, the concentration of free toxin and cell fate (growth, death, growth/death arrest and germination). Note, since only viable cells can transport nutrients and synthesize antitoxins, the intracellular nutrient concentration is a function of bulk nutrient concentration in the medium and the concentration of viable cells. Therefore, the simplest way to vary intracellular nutrient concentration is to vary the concentration of viable cells while keeping the bulk nutrient concentration constant (figure 4). If our model holds for free-living bacteria, we recommend that it also be tested in Archaea, which also are known to possess chromosomal TASs.

### 9.1. Predictions in rich media (i.e. tryptic soy broth with 0.6% yeast extract)

— When nutrients are in excess relative to viable cell density (population is below *K*), excess antitoxins are synthesized and thus all toxins are neutralized, which results in growth of vegetative cells. Note, the ratio of (nutrients)/(viable cells) determines the intracellular concentration of nutrients inside viable cells and can be altered by changing either the concentration of nutrients in the medium or the concentration of viable cells in the medium.
— When nutrients are limited relative to viable cell density (population is above *K*), low levels of antitoxins are synthesized and thus high levels of free toxin are rapidly released, which results in PCD.
— When nutrients are in equilibrium with viable cell density (population is at *K*), antitoxin synthesis is in equilibrium with antitoxin degradation; therefore, moderate and constant levels of antitoxin are synthesized and moderate and constant levels of free toxin are released, which results in growth/death arrest (dormancy).

### 9.2. Growth of the model organism *Listeria* in yeast extract broth

When *L. monocytogenes* or *Listeria innocua* are grown in yeast extract broth (YEB), which lacks Tryptone and Soytone (the main sources of amino acids found in TSBYE), the amino acid lysine becomes limited at high viable cell density, and the entire population of cells then dies rapidly (M Doan & SJ Knabel, unpublished observation, 2025). However, when dying cultures in YEB are spiked with lysine, death rapidly stops and growth resumes until the population reaches a *K* dependent on the concentration of lysine added (M Doan & SJ Knabel, unpublished observation, 2025). This may be due to the inability of cells to synthesize lysine and thus are unable to synthesize the lysine-containing antitoxin MazE when lysine becomes limited at high cell density. As a result, MazE would be rapidly reduced by constant degradation, and, thus high levels of the free toxin MazF would be rapidly released and subsequently cause PCD.

### 9.3. Predictions of *Listeria* in yeast extract broth

Addition of lysine to dying cells in YEB causes a rapid increase in synthesis of the antitoxin MazE, which neutralizes the toxin MazF, thereby stopping death and causing growth to resume. Addition of increasing concentrations of lysine to dying cells in YEB increases *K*, because when lysine is in excess, antitoxins are at first synthesized more rapidly than they are degraded, which neutralizes all toxins and results in growth. When the concentration of lysine is in equilibrium with the concentration of viable cells at *K*, the rate of antitoxin synthesis equals the rate of antitoxin degradation. This results in a moderate and constant level of free toxin, leading to growth arrest.

## Data Availability

This article has no additional data.

## References

[1] Burga A, Ben-David E, Kruglyak L. 2020 Toxin-antidote elements across the tree of life. Annu. Rev. Genet. **54**, 387–415. (10.1146/annurev-genet-112618-043659) 32886546

[2] Ogura T, Hiraga S. 1983 Mini-F plasmid genes that couple host cell division to plasmid proliferation. Proc. Natl Acad. Sci. USA **80**, 4784–4788. (10.1073/pnas.80.15.4784) 6308648 PMC384129

[3] Gerdes K, Rasmussen PB, Molin S. 1986 Unique type of plasmid maintenance function: postsegregational killing of plasmid-free cells. Proc. Natl Acad. Sci. USA **83**, 3116–3120. (10.1073/pnas.83.10.3116) 3517851 PMC323463

[4] Gerdes K, Christensen SK, Løbner-Olesen A. 2005 Prokaryotic toxin-antitoxin stress response loci. Nat. Rev. Microbiol. **3**, 371–382. (10.1038/nrmicro1147) 15864262

[5] Fraikin N, Goormaghtigh F, Van Melderen L. 2020 Type II toxin-antitoxin systems: evolution and revolutions. J. Bacteriol. **202**, e00763-19. (10.1128/jb.00763-19) 31932311 PMC7167474

[6] Shore SFH, Leinberger FH, Fozo EM, Berghoff BA. 2024 Type I toxin-antitoxin systems in bacteria: from regulation to biological functions. EcoSal Plus **12**, eesp00252022. (10.1128/ecosalplus.esp-0025-2022) 38767346 PMC11636113

[7] Buts L, Lah J, Dao-Thi MH, Wyns L, Loris R. 2005 Toxin-antitoxin modules as bacterial metabolic stress managers. Trends Biochem. Sci. **30**, 672–679. (10.1016/j.tibs.2005.10.004) 16257530

[8] Wagner EGH, Unoson C. 2012 The toxin-antitoxin system tisB-istR1. RNA Biol. **9**, 1513–1519. (10.4161/rna.22578) 23093802

[9] Frampton R, Aggio RBM, Villas-Bôas SG, Arcus VL, Cook GM. 2012 Toxin-antitoxin systems of Mycobacterium smegmatis are essential for cell survival. J. Biol. Chem. **287**, 5340–5356. (10.1074/jbc.m111.286856) 22199354 PMC3285314

[10] Arcus VL, Cook GM. 2013 Type II toxin-antitoxins: structural and functional aspects of type II loci in mycobacteria. In Prokaryotic toxin-antitoxins (ed. K Gerdes), pp. 137–156. Berlin, Germany: Springer Berlin Heidelberg. (10.1007/978-3-642-33253-1_8)

[11] Balaban NQ, Gerdes K, Lewis K, McKinney JD. 2013 A problem of persistence: still more questions than answers? Nat. Rev. Microbiol. **11**, 587–591. (10.1038/nrmicro3076) 24020075

[12] Fasani RA, Savageau MA. 2013 Molecular mechanisms of multiple toxin–antitoxin systems are coordinated to govern the persister phenotype. Proc. Natl Acad. Sci. USA **110**, E2528–37. (10.1073/pnas.1301023110) 23781105 PMC3703989

[13] Brantl S, Jahn N. 2015 sRNAs in bacterial type I and type III toxin-antitoxin systems. FEMS Microbiol. Rev. **39**, 413–427. (10.1093/femsre/fuv003) 25808661

[14] Chan WT, Espinosa M, Yeo CC. 2016 Keeping the wolves at bay: antitoxins of prokaryotic type II toxin-antitoxin systems. Front. Mol. Biosci. **3**, 9. (10.3389/fmolb.2016.00009) 27047942 PMC4803016

[15] Goormaghtigh F *et al*. 2018 Reassessing the role of type II toxin-antitoxin systems in formation of Escherichia coli Type II persister cells. MBio **9**, e00640-18. (10.1128/mBio.00640-18) 29895634 PMC6016239

[16] Harms A, Brodersen DE, Mitarai N, Gerdes K. 2018 Toxins, targets, and triggers: an overview of toxin-antitoxin biology. Mol. Cell **70**, 768–784. (10.1016/j.molcel.2018.01.003) 29398446

[17] Holden DW, Errington J. 2018 Type II toxin-antitoxin systems and persister cells. MBio **9**, e01574-18. (10.1128/mBio.01574-18) 30254124 PMC6156201

[18] Bordes P, Genevaux P. 2021 Control of toxin-antitoxin systems by proteases in Mycobacterium tuberculosis. Front. Mol. Biosci. **8**, 691399. (10.3389/fmolb.2021.691399) 34079824 PMC8165232

[19] Kamruzzaman M, Wu AY, Iredell JR. 2021 Biological functions of type II toxin-antitoxin systems in bacteria. Microorganisms **9**, 1276. (10.3390/microorganisms9061276) 34208120 PMC8230891

[20] De Bruyn P, Girardin Y, Loris R. 2021 Prokaryote toxin–antitoxin modules: complex regulation of an unclear function. Protein Sci. **30**, 1103–1113. (10.1002/pro.4071) 33786944 PMC8138530

[21] Singh G, Yadav M, Ghosh C, Rathore JS. 2021 Bacterial toxin-antitoxin modules: classification, functions, and association with persistence. Curr. Res. Microb. Sci. **2**, 100047. (10.1016/j.crmicr.2021.100047) 34841338 PMC8610362

[22] Song S, Wood TK. 2020 A primary physiological role of toxin/antitoxin systems is phage inhibition. Front. Microbiol. **11**, 1895. (10.3389/fmicb.2020.01895) 32903830 PMC7438911

[23] LeRoux M, Laub MT. 2022 Toxin-antitoxin systems as phage defense elements. Annu. Rev. Microbiol. **76**, 21–43. (10.1146/annurev-micro-020722-013730) 35395167

[24] Jain S, Bhowmick A, Jeong B, Bae T, Ghosh A. 2022 Unravelling the physiological roles of mazEF toxin–antitoxin system on clinical MRSA strain by CRISPR RNA-guided cytidine deaminase. J. Biomed. Sci. **29**, 28. (10.1186/s12929-022-00810-5) 35524246 PMC9077811

[25] Chan WT, Garcillán-Barcia MP, Yeo CC, Espinosa M. 2023 Type II bacterial toxin–antitoxins: hypotheses, facts, and the newfound plethora of the PezAT system. FEMS Microbiol. Rev. **47**, d052. (10.1093/femsre/fuad052) PMC10532202 37715317

[26] Pizzolato-Cezar LR, Spira B, Machini MT. 2023 Bacterial toxin-antitoxin systems: novel insights on toxin activation across populations and experimental shortcomings. Curr. Res. Microb. Sci. **5**, 100204. (10.1016/j.crmicr.2023.100204) 38024808 PMC10643148

[27] Magnuson RD. 2007 Hypothetical functions of toxin-antitoxin systems. J. Bacteriol. **189**, 6089–6092. (10.1128/jb.00958-07) 17616596 PMC1951896

[28] Wang X, Wood TK. 2011 Toxin-antitoxin systems influence biofilm and persister cell formation and the general stress response. Appl. Environ. Microbiol. **77**, 5577–5583. (10.1128/aem.05068-11) 21685157 PMC3165247

[29] Rosendahl S, Tamman H, Brauer A, Remm M, Hõrak R. 2020 Chromosomal toxin-antitoxin systems in Pseudomonas putida are rather selfish than beneficial. Sci. Rep. **10**, 9230. (10.1038/s41598-020-65504-0) 32513960 PMC7280312

[30] Kelly A, Arrowsmith TJ, Went SC, Blower TR. 2023 Toxin–antitoxin systems as mediators of phage defence and the implications for abortive infection. Curr. Opin. Microbiol. **73**, 102293. (10.1016/j.mib.2023.102293) 36958122

[31] Aizenman E, Engelberg-Kulka H, Glaser G. 1996 An Escherichia coli chromosomal ‘addiction module’ regulated by guanosine [corrected] 3’,5’-bispyrophosphate: a model for programmed bacterial cell death. Proc. Natl Acad. Sci. USA **93**, 6059–6063. (10.1073/pnas.93.12.6059) 8650219 PMC39188

[32] Bergkessel M, Basta DW, Newman DK. 2016 The physiology of growth arrest: uniting molecular and environmental microbiology. Nat. Rev. Microbiol. **14**, 549–562. (10.1038/nrmicro.2016.107) 27510862 PMC10069271

[33] Efeyan A, Comb WC, Sabatini DM. 2015 Nutrient-sensing mechanisms and pathways. Nature **517**, 302–310. (10.1038/nature14190) 25592535 PMC4313349

[34] Dworkin J, Losick R. 2001 Differential gene expression governed by chromosomal spatial asymmetry. Cell **107**, 339–346. (10.1016/s0092-8674(01)00528-1) 11701124

[35] Patnaik R. 2008 Engineering complex phenotypes in industrial strains. Biotechnol. Prog. **24**, 38–47. (10.1021/bp0701214) 17914860

[36] Ramkrishna D. 1983 A cybernetic perspective of microbial growth. In Foundations of biochemical engineering ACS symposium series (eds HW Blanch, T Papoutsakis, G Stephanopoulos), pp. 161–178. Washington, DC: American Chemical Society. (10.1021/bk-1983-0207.ch007)

[37] Stebbing T. 2010 A cybernetic view of biological growth: the Maia hypothesis. Cambridge, UK: Cambridge University Press. (10.1017/CBO9780511933813)

[38] Cappuccino N, Price PW (eds). 1995 Population dynamics: new approaches and synthesis. San Diego, CA: Academic Press.

[39] Kędzierska B, Hayes F. 2016 Emerging roles of toxin-antitoxin modules in bacterial pathogenesis. Molecules **21**, 790. (10.3390/molecules21060790) 27322231 PMC6273597

[40] Li GW, Burkhardt D, Gross C, Weissman JS. 2014 Quantifying absolute protein synthesis rates reveals principles underlying allocation of cellular resources. Cell **157**, 624–635. (10.1016/j.cell.2014.02.033) 24766808 PMC4006352

[41] Deter H, Jensen R, Mather W, Butzin N. 2017 Mechanisms for differential protein production in toxin–antitoxin systems. Toxins **9**, 211. (10.3390/toxins9070211) 28677629 PMC5535158

[42] Hazan R, Sat B, Engelberg-Kulka H. 2004 Escherichia coli mazEF-mediated cell death is triggered by various stressful conditions. J. Bacteriol. **186**, 3663–3669. (10.1128/JB.186.11.3663-3669.2004) 15150257 PMC415763

[43] Mittenhuber G. 1999 Occurrence of mazEF-like antitoxin/toxin systems in bacteria. J. Mol. Microbiol. Biotechnol. **1**, 295–302. 10943559

[44] Yamaguchi Y, Park JH, Inouye M. 2011 Toxin-antitoxin systems in bacteria and archaea. Annu. Rev. Genet. **45**, 61–79. (10.1146/annurev-genet-110410-132412) 22060041

[45] Metzger S, Dror IB, Aizenman E, Schreiber G, Toone M, Friesen JD, Cashel M, Glaser G. 1988 The nucleotide sequence and characterization of the relA gene of Escherichia coli. J. Biol. Chem. **263**, 15699–15704. (10.1016/s0021-9258(19)37644-6) 2844820

[46] Weber H, Polen T, Heuveling J, Wendisch VF, Hengge R. 2005 Genome-wide analysis of the general stress response network in Escherichia coli: σ^S^-dependent genes, promoters, and sigma factor selectivity. J. Bacteriol. **187**, 1591–1603. (10.1128/JB.187.5.1591-1603.2005) 15716429 PMC1063999

[47] Battesti A, Majdalani N, Gottesman S. 2011 The RpoS-mediated general stress response in Escherichia coli. Annu. Rev. Microbiol. **65**, 189–213. (10.1146/annurev-micro-090110-102946) 21639793 PMC7356644

[48] Schuster CF, Bertram R. 2013 Toxin-antitoxin systems are ubiquitous and versatile modulators of prokaryotic cell fate. FEMS Microbiol. Lett. **340**, 73–85. (10.1111/1574-6968.12074) 23289536

[49] Donegan NP, Cheung AL. 2009 Regulation of the mazEF toxin-antitoxin module in Staphylococcus aureus and its impact on sigB expression. J. Bacteriol. **191**, 2795–2805. (10.1128/jb.01713-08) 19181798 PMC2668418

[50] Gerdes K. 2000 Toxin-antitoxin modules may regulate synthesis of macromolecules during nutritional stress. J. Bacteriol. **182**, 561–572. (10.1128/jb.182.3.561-572.2000) 10633087 PMC94316

[51] Christensen SK, Mikkelsen M, Pedersen K, Gerdes K. 2001 RelE, a global inhibitor of translation, is activated during nutritional stress. Proc. Natl Acad. Sci. USA **98**, 14328–14333. (10.1073/pnas.251327898) 11717402 PMC64681

[52] Ramisetty BCM. 2020 Regulation of type II toxin-antitoxin systems: the translation-responsive model. Front. Microbiol. **11**, 895. (10.3389/fmicb.2020.00895) 32431690 PMC7214741

[53] Ostyn E, Augagneur Y, Pinel-Marie ML. 2025 Insight into the environmental cues modulating the expression of bacterial toxin-antitoxin systems. FEMS Microbiol. Rev. **49**, f007. (10.1093/femsre/fuaf007) PMC11951105 40052347

[54] LeRoux M, Culviner PH, Liu YJ, Littlehale ML, Laub MT. 2020 Stress can induce transcription of toxin-antitoxin systems without activating toxin. Mol. Cell **79**, 280–292.(10.1016/j.molcel.2020.05.028) 32533919 PMC7368831

[55] Engelberg-Kulka H, Amitai S, Kolodkin-Gal I, Hazan R. 2006 Bacterial programmed cell death and multicellular behavior in bacteria. PLoS Genet. **2**, e135. (10.1371/journal.pgen.0020135) 17069462 PMC1626106

[56] Hall AM, Gollan B, Helaine S. 2017 Toxin–antitoxin systems: reversible toxicity. Curr. Opin. Microbiol. **36**, 102–110. (10.1016/j.mib.2017.02.003) 28279904

[57] Tompa P. 2002 Intrinsically unstructured proteins. Trends Biochem. Sci. **27**, 527–533. (10.1016/s0968-0004(02)02169-2) 12368089

[58] Wright PE, Dyson HJ. 1999 Intrinsically unstructured proteins: re-assessing the protein structure-function paradigm. J. Mol. Biol. **293**, 321–331. (10.1006/jmbi.1999.3110) 10550212

[59] Loris R, Garcia-Pino A. 2014 Disorder- and dynamics-based regulatory mechanisms in toxin–antitoxin modules. Chem. Rev. **114**, 6933–6947. (10.1021/cr400656f) 24806488

[60] De Gieter S, Konijnenberg A, Talavera A, Butterer A, Haesaerts S, De Greve H, Sobott F, Loris R, Garcia-Pino A. 2014 The intrinsically disordered domain of the antitoxin Phd chaperones the toxin Doc against irreversible inactivation and misfolding. J. Biol. Chem. **289**, 34013–34023. (10.1074/jbc.m114.572396) 25326388 PMC4256337

[61] Maisonneuve E, Castro-Camargo M, Gerdes K. 2013 Retracted: (p)ppGpp controls bacterial persistence by stochastic induction of toxin-antitoxin activity. Cell **154**, 1140–1150. (10.1016/j.cell.2013.07.048) 23993101

[62] Tsilibaris V, Maenhaut-Michel G, Mine N, Van Melderen L. 2007 What is the benefit to Escherichia coli of having multiple toxin-antitoxin systems in its genome? J. Bacteriol. **189**, 6101–6108. (10.1128/jb.00527-07) 17513477 PMC1951899

[63] Van Melderen L, Aertsen A. 2009 Regulation and quality control by Lon-dependent proteolysis. Res. Microbiol. **160**, 645–651. (10.1016/j.resmic.2009.08.021) 19772918

[64] Brzozowska I, Zielenkiewicz U. 2013 Regulation of toxin–antitoxin systems by proteolysis. Plasmid **70**, 33–41. (10.1016/j.plasmid.2013.01.007) 23396045

[65] Ramisetty BCM. 2015 Regulation of yefM/yoeB toxin antitoxin system is independent of ppGpp and inorganic polyphosphate in Escherichia coli. bioRxiv 021162. (10.1101/021162)

[66] Christensen SK, Maenhaut-Michel G, Mine N, Gottesman S, Gerdes K, Van Melderen L. 2004 Overproduction of the Lon protease triggers inhibition of translation in Escherichia coli: involvement of the yefM-yoeB toxin-antitoxin system. Mol. Microbiol. **51**, 1705–1717. (10.1046/j.1365-2958.2003.03941.x) 15009896

[67] Gelens L, Hill L, Vandervelde A, Danckaert J, Loris R. 2013 A general model for toxin-antitoxin module dynamics can explain persister cell formation in E. coli. PLoS Comput. Biol. **9**, e1003190. (10.1371/journal.pcbi.1003190) 24009490 PMC3757116

[68] Lioy VS *et al*. 2006 pSM19035-encoded zeta toxin induces stasis followed by death in a subpopulation of cells. Microbiology **152**, 2365–2379. (10.1099/mic.0.28950-0) 16849801

[69] Edelmann D, Berghoff BA. 2022 A shift in perspective: a role for the type I toxin TisB as persistence-stabilizing factor. Front. Microbiol. **13**, 871699. (10.3389/fmicb.2022.871699) 35369430 PMC8969498

[70] Vogel J, Argaman L, Wagner EGH, Altuvia S. 2004 The small RNA IstR inhibits synthesis of an SOS-induced toxic peptide. Curr. Biol. **14**, 2271–2276. (10.1016/j.cub.2004.12.003) 15620655

[71] Wen J, Fozo E. 2014 sRNA antitoxins: more than one way to repress a toxin. Toxins **6**, 2310–2335. (10.3390/toxins6082310) 25093388 PMC4147584

[72] Tsatsakis A *et al*. 2017 Simulating real-life exposures to uncover possible risks to human health: a proposed consensus for a novel methodological approach. Hum. Exp. Toxicol. **36**, 554–564. (10.1177/0960327116681652) 28539089

[73] Vesper O, Amitai S, Belitsky M, Byrgazov K, Kaberdina AC, Engelberg-Kulka H, Moll I. 2011 Selective translation of leaderless mRNAs by specialized ribosomes generated by MazF in Escherichia coli. Cell **147**, 147–157. (10.1016/j.cell.2011.07.047) 21944167 PMC4894548

[74] Nigam A, Ziv T, Oron-Gottesman A, Engelberg-Kulka H. 2019 Stress-induced MazF-mediated proteins in Escherichia coli. mBio **10**, e00340-19. (10.1128/mbio.00340-19) 30914510 PMC6437054

[75] Nikolic N. 2019 Autoregulation of bacterial gene expression: lessons from the MazEF toxin–antitoxin system. Curr. Genet. **65**, 133–138. (10.1007/s00294-018-0879-8) 30132188 PMC6343021

[76] Hobby GL, Meyer K, Chaffee E. 1942 Observations on the mechanism of action of penicillin. Exp. Biol. Med. **50**, 281–285. (10.3181/00379727-50-13773)

[77] Bigger JW. 1944 Treatment of staphylococcal infections with penicillin by intermittent sterilisation. Lancet **244**, 497–500. (10.1016/s0140-6736(00)74210-3)

[78] Wood TK, Knabel SJ, Kwan BW. 2013 Bacterial persister cell formation and dormancy. Appl. Environ. Microbiol. **79**, 7116–7121. (10.1128/aem.02636-13) 24038684 PMC3837759

[79] Balaban NQ, Merrin J, Chait R, Kowalik L, Leibler S. 2004 Bacterial persistence as a phenotypic switch. Science **305**, 1622–1625. (10.1126/science.1099390) 15308767

[80] Thakur Z, Chaudhary R, Mehta PK. 2024 Deciphering the role of VapBC toxin-antitoxin systems in Mycobacterium tuberculosis stress adaptation. Future Microbiol. **19**, 1587–1599. (10.1080/17460913.2024.2412447) 39431307

[81] Van Melderen L. 2010 Toxin–antitoxin systems: why so many, what for? Curr. Opin. Microbiol. **13**, 781–785. (10.1016/j.mib.2010.10.006) 21041110

[82] Harrison JJ, Wade WD, Akierman S, Vacchi-Suzzi C, Stremick CA, Turner RJ, Ceri H. 2009 The chromosomal toxin gene yafQ is a determinant of multidrug tolerance for Escherichia coli growing in a biofilm. Antimicrob. Agents Chemother. **53**, 2253–2258. (10.1128/aac.00043-09) 19307375 PMC2687228

[83] Kim Y, Wood TK. 2010 Toxins Hha and CspD and small RNA regulator Hfq are involved in persister cell formation through MqsR in Escherichia coli. Biochem. Biophys. Res. Commun. **391**, 209–213. (10.1016/j.bbrc.2009.11.033) 19909729 PMC2812665

[84] Lewis K. 2010 Persister cells. Annu. Rev. Microbiol. **64**, 357–372. (10.1146/annurev.micro.112408.134306) 20528688

[85] Keren I, Shah D, Spoering A, Kaldalu N, Lewis K. 2004 Specialized persister cells and the mechanism of multidrug tolerance in Escherichia coli. J. Bacteriol. **186**, 8172–8180. (10.1128/JB.186.24.8172-8180.2004) 15576765 PMC532439

[86] Fishov I, Zaritsky A, Grover NB. 1995 On microbial states of growth. Mol. Microbiol. **15**, 789–794. (10.1111/j.1365-2958.1995.tb02349.x) 7596281

[87] Dawes IW, Mandelstam J. 1970 Sporulation of Bacillus subtilis in continuous culture. J. Bacteriol. **103**, 529–535. (10.1128/jb.103.3.529-535.1970) 4990846 PMC248122

[88] Sanchez-Torres V, Hwang HJ, Wood TK. 2024 Conformational change as a mechanism for toxin activation in bacterial toxin-antitoxin systems. J. Virol. **98**, e0151324. (10.1128/jvi.01513-24) 39445801 PMC11575165

[89] Allocati N, Masulli M, Di Ilio C, De Laurenzi V. 2015 Die for the community: an overview of programmed cell death in bacteria. Cell Death Dis. **6**, e1609–e1609. (10.1038/cddis.2014.570) 25611384 PMC4669768

[90] Tanouchi Y, Pai A, Buchler NE, You L. 2012 Programming stress‐induced altruistic death in engineered bacteria. Mol. Syst. Biol. **8**, 626. (10.1038/msb.2012.57) 23169002 PMC3531911

[91] Bayles KW. 2014 Bacterial programmed cell death: making sense of a paradox. Nat. Rev. Microbiol. **12**, 63–69. (10.1038/nrmicro3136) 24336185 PMC4422510

[92] Peeters SH, de Jonge MI. 2018 For the greater good: programmed cell death in bacterial communities. Microbiol. Res. **207**, 161–169. (10.1016/j.micres.2017.11.016) 29458850

[93] Kulkarni M, Hardwick JM. 2023 Programmed cell death in unicellular versus multicellular organisms. Annu. Rev. Genet. **57**, 435–459. (10.1146/annurev-genet-033123-095833) 37722687 PMC11491101

[94] Nieto C, Cherny I, Khoo SK, de Lacoba MG, Chan WT, Yeo CC, Gazit E, Espinosa M. 2007 The yefM-yoeB toxin-antitoxin systems of Escherichia coli and Streptococcus pneumoniae: functional and structural correlation. J. Bacteriol. **189**, 1266–1278. (10.1128/JB.01130-06) 17071753 PMC1797350

[95] Fu Z, Tamber S, Memmi G, Donegan NP, Cheung AL. 2009 Overexpression of MazF [sub]Sa[/sub] in Staphylococcus aureus induces bacteriostasis by selectively targeting mRNAs for cleavage. J. Bacteriol. **191**, 2051–2059. (10.1128/jb.00907-08) 19168622 PMC2655526

[96] Pedersen K, Christensen SK, Gerdes K. 2002 Rapid induction and reversal of a bacteriostatic condition by controlled expression of toxins and antitoxins. Mol. Microbiol. **45**, 501–510. (10.1046/j.1365-2958.2002.03027.x) 12123459

[97] Palmer T, Bonner PL. 2011 Enzyme inhibition. In Enzymes: biochemistry, biotechnology, clinical chemistry (eds T Palmer, PL Bonner), pp. 126–152, 2nd edn. Cambridge, UK: Woodhead Publishing. (10.1533/9780857099921.2.126)

[98] Engelberg-Kulka H, Glaser G. 1999 Addiction modules and programmed cell death and antideath in bacterial cultures. Annu. Rev. Microbiol. **53**, 43–70. (10.1146/annurev.micro.53.1.43) 10547685

[99] Pedersen K, Gerdes K. 1999 Multiple hok genes on the chromosome of Escherichia coli. Mol. Microbiol. **32**, 1090–1102. (10.1046/j.1365-2958.1999.01431.x) 10361310

[100] Amitai S, Yassin Y, Engelberg-Kulka H. 2004 MazF-mediated cell death in Escherichia coli: a point of no return. J. Bacteriol. **186**, 8295–8300. (10.1128/JB.186.24.8295-8300.2004) 15576778 PMC532418

[101] Lah J, Šimić M, Vesnaver G, Marianovsky I, Glaser G, Engelberg-Kulka H, Loris R. 2005 Energetics of structural transitions of the addiction antitoxin MazE: is a programmed bacterial cell death dependent on the intrinsically flexible nature of the antitoxins? J. Biol. Chem. **280**, 17397–17407. (10.1074/jbc.M501128200) 15735309

[102] Yamaguchi Y, Inouye M. 2011 Regulation of growth and death in Escherichia coli by toxin–antitoxin systems. Nat. Rev. Microbiol. **9**, 779–790. (10.1038/nrmicro2651) 21927020

[103] Sat B, Reches M, Engelberg-Kulka H. 2003 The Escherichia coli mazEF suicide module mediates thymineless death. J. Bacteriol. **185**, 1803–1807. (10.1128/jb.185.6.1803-1807.2003) 12618443 PMC150121

[104] Kwan BW, Valenta JA, Benedik MJ, Wood TK. 2013 Arrested protein synthesis increases persister-like cell formation. Antimicrob. Agents Chemother. **57**, 1468–1473. (10.1128/aac.02135-12) 23295927 PMC3591907

[105] Hazan R, Sat B, Reches M, Engelberg-Kulka H. 2001 Postsegregational killing mediated by the P1 phage ‘addiction module’ phd-doc requires the Escherichia coli programmed cell death system mazEF. J. Bacteriol. **183**, 2046–2050. (10.1128/JB.183.6.2046-2050.2001) 11222604 PMC95101

[106] Korch SB, Hill TM. 2006 Ectopic overexpression of wild-type and mutant hipA genes in Escherichia coli: effects on macromolecular synthesis and persister formation. J. Bacteriol. **188**, 3826–3836. (10.1128/JB.01740-05) 16707675 PMC1482909

[107] Jimmy S *et al*. 2020 A widespread toxin−antitoxin system exploiting growth control via alarmone signaling. Proc. Natl Acad. Sci. USA **117**, 10500–10510. (10.1073/pnas.1916617117) 32345719 PMC7229694

[108] Kaldalu N, Hauryliuk V, Tenson T. 2016 Persisters—as elusive as ever. Appl. Microbiol. Biotechnol. **100**, 6545–6553. (10.1007/s00253-016-7648-8) 27262568 PMC4939303

[109] Pandey DP, Gerdes K. 2005 Toxin-antitoxin loci are highly abundant in free-living but lost from host-associated prokaryotes. Nucleic Acids Res. **33**, 966–976. (10.1093/nar/gki201) 15718296 PMC549392

[110] Hamilton WD. 1964 The genetical evolution of social behaviour. I. J. Theor. Biol. **7**, 1–16. (10.1016/0022-5193(64)90038-4) 5875341

[111] West SA, Gardner A. 2013 Adaptation and inclusive fitness. Curr. Biol. **23**, R577–R584. (10.1016/j.cub.2013.05.031) 23845249

[112] Prozorov AA, Danilenko VN. 2010 Toxin-antitoxin systems in bacteria: apoptotic tools or metabolic regulators? Microbiology **79**, 129–140. (10.1134/s0026261710020013)20411670

[113] Shapiro JA. 1988 Bacteria as multicellular organisms. Sci. Am. **258**, 82–89. (10.1038/scientificamerican0688-82)2847312

[114] Malthus T. 1798 An essay on the principle of population, as it affects the future improvement of society, with remarks on the speculations of Mr. Godwin, M. Condorcet, and other writers, 1st edn. London, UK: J. Johnson.

[115] Odum EP. 1953 Fundamentals of ecology. Philadelphia, PA: W.B. Saunders Company.

[116] Verhulst PF. 1838 Notice on the law that a population follows in its growth. Corr. Math. Phys. **10**, 113–121.

[117] Hutchinson GE. 1948 Circular causal systems in ecology. Ann. NY Acad. Sci. **50**, 221–246. (10.1111/j.1749-6632.1948.tb39854.x) 18886382

[118] Darwin C, Wallace A. 1858 On the Tendency of species to form varieties; and on the perpetuation of varieties and species by natural means of selection. Zool. J. Linn. Soc. **3**, 45–62. (10.1111/j.1096-3642.1858.tb02500.x)

[119] MacArthur RH, Wilson EO. 1967 The theory of island biogeography. Princeton, NJ: Princeton University Press. See https://www.jstor.org/stable/j.ctt19cc1t2.

[120] Pianka ER. 1970 On r- and K-selection. Am. Nat. **104**, 592–597. (10.1086/282697)

[121] Brommer JE. 2000 The evolution of fitness in life-history theory. Biol. Rev. Camb. Philos. Soc. **75**, 377–404. (10.1017/s000632310000551x) 11034016

[122] Huang M, Hull CM. 2017 Sporulation: how to survive on planet Earth (and beyond). Curr. Genet. **63**, 831–838. (10.1007/s00294-017-0694-7) 28421279 PMC5647196

[123] Losick R, Youngman P, Piggot PJ. 1986 Genetics of endospore formation in Bacillus subtilis. Annu. Rev. Genet. **20**, 625–669. (10.1146/annurev.ge.20.120186.003205) 3101583

[124] Cano RJ, Borucki MK. 1995 Revival and identification of bacterial spores in 25- to 40-million-year-old Dominican amber. Science **268**, 1060–1064. (10.1126/science.7538699) 7538699

[125] Reder A, Gerth U, Hecker M. 2012 Integration of σ^B^ activity into the decision-making process of sporulation initiation in Bacillus subtilis. J. Bacteriol. **194**, 1065–1074. (10.1128/jb.06490-11) 22210769 PMC3294812

[126] Ayala FR, Bartolini M, Grau R. 2020 The stress-responsive alternative sigma factor SigB of Bacillus subtilis and its relatives: an old friend with new functions. Front. Microbiol. **11**, 1761. (10.3389/fmicb.2020.01761) 33042030 PMC7522486

[127] Yeak KYC *et al*. 2023 SigB modulates expression of novel SigB regulon members via Bc1009 in non-stressed and heat-stressed cells revealing its alternative roles in Bacillus cereus. BMC Microbiol. **23**, 37. (10.1186/s12866-023-02783-3) 36759782 PMC9912610

[128] Zhao Z, Hajiahmadi F, Alehashem MS, Williams AH. 2024 Molecular architecture and function of the bacterial stressosome. Curr. Opin. Microbiol. **82**, 102541. (10.1016/j.mib.2024.102541) 39270610

[129] Nicholson WL. 2002 Roles of Bacillus endospores in the environment. Cell. Mol. Life Sci. **59**, 410–416. (10.1007/s00018-002-8433-7) 11964119 PMC11337551

[130] Gottesman S. 2019 Trouble is coming: signaling pathways that regulate general stress responses in bacteria. J. Biol. Chem. **294**, 11685–11700. (10.1074/jbc.rev119.005593) 31197038 PMC6682744

[131] Drews G. 2000 The roots of microbiology and the influence of Ferdinand Cohn on microbiology of the 19th century. FEMS Microbiol. Rev. **24**, 225–249. (10.1016/s0168-6445(00)00026-7) 10841971

[132] Piggot PJ, Losick R. 2001 Sporulation genes and intercompartmental regulation. In Bacillus subtilis and its closest relatives (eds AL Sonenshein, JA Hoch, R Losick), pp. 483–517. New York, NY: John Wiley & Sons, Ltd. (10.1128/9781555817992.ch34)

[133] Gould GW, Dring GJ. 1975 Heat resistance of bacterial endospores and concept of an expanded osmoregulatory cortex. Nature **258**, 402–405. (10.1038/258402a0) 1196370

[134] Moir A, Cooper G. 2015 Spore germination. Microbiol. Spectr. **3** 1–19. . (10.1128/microbiolspec.tbs-0014-2012) 27337279

[135] Beskrovnaya P, Sexton DL, Golmohammadzadeh M, Hashimi A, Tocheva EI. 2021 Structural, metabolic and evolutionary comparison of bacterial endospore and exospore formation. Front. Microbiol. **12**, 630573. (10.3389/fmicb.2021.630573) 33767680 PMC7985256

[136] Corona Ramírez A, Lee KS, Odriozola A, Kaminek M, Stocker R, Zuber B, Junier P. 2023 Multiple roads lead to Rome: unique morphology and chemistry of endospores, exospores, myxospores, cysts and akinetes in bacteria. Microbiology **169**, 001299. (10.1099/mic.0.001299) 36804869 PMC10197873

[137] Trujillo ME, Dedysh S, DeVos P, Hedlund B, Kämpfer P, Rainey FA, Whitman WB (eds). 2015 Bergey’s manual of systematics of archaea and bacteria, 1st edn. Hoboken, NJ: Wiley. (10.1002/9781118960608)

[138] Morita RY. 1975 Psychrophilic bacteria. Bacteriol. Rev. **39**, 144–167. (10.1128/br.39.2.144-167.1975) 1095004 PMC413900

[139] Morita RY. 1993 Bioavailability of energy and the starvation state. In Starvation in bacteria (ed. S Kjelleberg), pp. 1–23. Boston, MA, USA: Springer US. (10.1007/978-1-4899-2439-1_1)

[140] Kjelleberg S (ed). 1993 Starvation in bacteria. Boston, MA, USA: Springer US. (10.1007/978-1-4899-2439-1)

[141] Lange R, Hengge-Aronis R. 1991 Growth phase-regulated expression of bolA and morphology of stationary-phase Escherichia coli cells are controlled by the novel sigma factor sigma S. J. Bacteriol. **173**, 4474–4481. (10.1128/jb.173.14.4474-4481.1991) 1648559 PMC208111

[142] Traxler MF, Summers SM, Nguyen H, Zacharia VM, Hightower GA, Smith JT, Conway T. 2008 The global, ppGpp‐mediated stringent response to amino acid starvation in Escherichia coli. Mol. Microbiol. **68**, 1128–1148. (10.1111/j.1365-2958.2008.06229.x) 18430135 PMC3719176

[143] Wen J, Anantheswaran RC, Knabel SJ. 2009 Changes in barotolerance, thermotolerance, and cellular morphology throughout the life cycle of Listeria monocytogenes. Appl. Environ. Microbiol. **75**, 1581–1588. (10.1128/aem.01942-08) 19168646 PMC2655472

[144] Fernández-Martínez LT, Javelle A, Hoskisson PA. 2024 Microbial primer: bacterial growth kinetics. Microbiology **170**, 001428. (10.1099/mic.0.001428) 38329407 PMC10924458

[145] Henrici AT. 1928 Morphologic variation and the rate of growth of bacteria. Springfield, IL: C.C. Thomas. (10.5962/bhl.title.7269)

[146] Shah D, Zhang Z, Khodursky A, Kaldalu N, Kurg K, Lewis K. 2006 Persisters: a distinct physiological state of E. coli. BMC Microbiol. **6**, 53. (10.1186/1471-2180-6-53) 16768798 PMC1557402

[147] Buchanan RE. 1918 Life phases in a bacterial culture. J. Infect. Dis. **23**, 109–125. (10.1086/infdis/23.2.109)

[148] Kurath G, Morita RY. 1983 Starvation-survival physiological studies of a marine Pseudomonas sp. Appl. Environ. Microbiol. **45**, 1206–1211. (10.1128/aem.45.4.1206-1211.1983) 16346265 PMC242440

[149] Barry K, Mendonça A, Phillips GJ, Boylston T, Fortes-Da-Silva P, Brehm-Stecher B, Juneja V, Wan Z. 2024 Long-term-survival phase cells of Salmonella enteritidis ATCC 13076 exhibit significantly greater tolerance to atmospheric cold plasma treatment of shell eggs. Front. Food. Sci. Technol. **4**, 1442761. (10.3389/frfst.2024.1442761)

[150] Bhullar MS, Shaw A, Mendonca A, Monge A, Nabwire L, Thomas-Popo E. 2021 Shiga toxin–producing Escherichia coli in the long-term survival phase exhibit higher chlorine tolerance and less sublethal injury following chlorine treatment of romaine lettuce. Foodborne Pathog. Dis. **18**, 276–282. (10.1089/fpd.2020.2873) 33471590

[151] Hengge-Aronis R. 2002 Signal transduction and regulatory mechanisms involved in control of the σ^S^ (RpoS) subunit of RNA polymerase. Microbiol. Mol. Biol. Rev. **66**, 373–395. (10.1128/mmbr.66.3.373-395.2002) 12208995 PMC120795

[152] Lee EJ, Karoonuthaisiri N, Kim HS, Park JH, Cha CJ, Kao CM, Roe JH. 2005 A master regulator σ^B^ governs osmotic and oxidative response as well as differentiation via a network of sigma factors in Streptomyces coelicolor. Mol. Microbiol. **57**, 1252–1264. (10.1111/j.1365-2958.2005.04761.x) 16101999

[153] Hirsch CF, Ensign JC. 1976 Heat activation of Streptomyces viridochromogenes spores. J. Bacteriol. **126**, 24–30. (10.1128/jb.126.1.24-30.1976) 4424 PMC233255

[154] Ma C, Sim S, Shi W, Du L, Xing D, Zhang Y. 2010 Energy production genes sucB and ubiF are involved in persister survival and tolerance to multiple antibiotics and stresses in Escherichia coli. FEMS Microbiol. Lett. **303**, 33–40. (10.1111/j.1574-6968.2009.01857.x) 20041955

[155] Amato SM, Orman MA, Brynildsen MP. 2013 Metabolic control of persister formation in Escherichia coli. Mol. Cell **50**, 475–487. (10.1016/j.molcel.2013.04.002) 23665232

[156] Prax M, Bertram R. 2014 Metabolic aspects of bacterial persisters. Front. Cell. Infect. Microbiol. **4**, 148. (10.3389/fcimb.2014.00148) 25374846 PMC4205924

[157] Semanjski M *et al*. 2021 Proteome dynamics during antibiotic persistence and resuscitation. mSystems **6**, e00549-21. (10.1128/msystems.00549-21) 34427514 PMC8407246

[158] Lyons NA, Kolter R. 2015 On the evolution of bacterial multicellularity. Curr. Opin. Microbiol. **24**, 21–28. (10.1016/j.mib.2014.12.007) 25597443 PMC4380822

[159] Penesyan A, Paulsen IT, Kjelleberg S, Gillings MR. 2021 Three faces of biofilms: a microbial lifestyle, a nascent multicellular organism, and an incubator for diversity. Npj Biofilms Microbiomes **7**, 9. (10.1038/s41522-021-00251-2) 34759294 PMC8581019

[160] Rice KC, Bayles KW. 2003 Death’s toolbox: examining the molecular components of bacterial programmed cell death. Mol. Microbiol. **50**, 729–738. (10.1046/j.1365-2958.2003.t01-1-03720.x) 14617136

[161] Wen J, Karthikeyan S, Hawkins J, Anantheswaran RC, Knabel SJ. 2013 Listeria monocytogenes responds to cell density as it transitions to the long-term-survival phase. Int. J. Food Microbiol. **165**, 326–331. (10.1016/j.ijfoodmicro.2013.05.014) 23810956

[162] Pardee AB, Prestidge LS. 1955 Induced formation of serine and threonine deaminases by Escherichia coli. J. Bacteriol. **70**, 667–674. (10.1128/jb.70.6.667-674.1955) 13271312 PMC386270

[163] Holland JH. 1999 Emergence: from chaos to order. Oxford, UK: Oxford University Press.

[164] Holland JH. 1992 Complex adaptive systems. Daedalus **121**, 17–30.

[165] Wiener N. 1948 Cybernetics or control and communication in the animal and the machine. Cambridge, MA: The MIT Press.

[166] Dhurjati P, Ramkrishna D, Flickinger MC, Tsao GT. 1985 A cybernetic view of microbial growth: modeling of cells as optimal strategists. Biotechnol. Bioeng. **27**, 1–9. (10.1002/bit.260270102) 18553570

[167] Young JD. 2015 Learning from the steersman: a natural history of cybernetic models. Ind. Eng. Chem. Res. **54**, 10162–10169. (10.1021/acs.iecr.5b01315)

[168] Cherny I, Gazit E. 2004 The YefM antitoxin defines a family of natively unfolded proteins. J. Biol. Chem. **279**, 8252–8261. (10.1074/jbc.m308263200) 14672926

[169] McCutcheon JP, Moran NA. 2010 Functional convergence in reduced genomes of bacterial symbionts spanning 200 My of evolution. Genome Biol. Evol. **2**, 708–718. (10.1093/gbe/evq055) 20829280 PMC2953269

[170] Cashel M, Gallant J. 1969 Two compounds implicated in the function of the RC gene of Escherichia coli. Nature **221**, 838–841. (10.1038/221838a0) 4885263

[171] Potrykus K, Cashel M. 2008 (p)ppGpp: still magical? Annu. Rev. Microbiol. **62**, 35–51. (10.1146/annurev.micro.62.081307.162903) 18454629

[172] Zhu M, Dai X. 2023 Stringent response ensures the timely adaptation of bacterial growth to nutrient downshift. Nat. Commun. **14**, 467. (10.1038/s41467-023-36254-0) 36709335 PMC9884231

[173] Winther KS, Roghanian M, Gerdes K. 2018 Activation of the stringent response by loading of RelA-tRNA complexes at the ribosomal A-site. Mol. Cell **70**, 95–105.(10.1016/j.molcel.2018.02.033) 29625042

[174] Hengge R. 2011 Stationary-phase gene regulation in Escherichia coli. EcoSal Plus **4**, 2176. (10.1128/ecosalplus.5.6.3) 26442507

[175] Dufour A, Haldenwang WG. 1994 Interactions between a Bacillus subtilis anti-sigma factor (RsbW) and its antagonist (RsbV). J. Bacteriol. **176**, 1813–1820. (10.1128/jb.176.7.1813-1820.1994) 8144446 PMC205282

[176] Guerreiro DN, Arcari T, O’Byrne CP. 2020 The σ^B^-mediated general stress response of Listeria monocytogenes: life and death decision making in a pathogen. Front. Microbiol. **11**, 1505. (10.3389/fmicb.2020.01505) 32733414 PMC7358398

[177] Benson AK, Haldenwang WG. 1993 Bacillus subtilis sigma B is regulated by a binding protein (RsbW) that blocks its association with core RNA polymerase. Proc. Natl Acad. Sci. USA **90**, 2330–2334. (10.1073/pnas.90.6.2330) 8460143 PMC46080

[178] Hardwick SW, Pané-Farré J, Delumeau O, Marles-Wright J, Murray JW, Hecker M, Lewis RJ. 2007 Structural and functional characterization of partner switching regulating the environmental stress response in Bacillus subtilis. J. Biol. Chem. **282**, 11562–11572. (10.1074/jbc.M609733200) 17303566

[179] Paget M. 2015 Bacterial sigma factors and anti-sigma factors: structure, function and distribution. Biomolecules **5**, 1245–1265. (10.3390/biom5031245) 26131973 PMC4598750

[180] Narula J, Tiwari A, Igoshin OA. 2016 Role of autoregulation and relative synthesis of operon partners in alternative sigma factor networks. PLoS Comput. Biol. **12**, e1005267. (10.1371/journal.pcbi.1005267) 27977677 PMC5207722

[181] Bouillet S, Hamdallah I, Majdalani N, Tripathi A, Gottesman S. 2024 A negative feedback loop is critical for recovery of RpoS after stress in Escherichia coli. PLoS Genet. **20**, e1011059. (10.1371/journal.pgen.1011059) 38466775 PMC10957080

[182] Harms M *et al*. 2024 Activation of the general stress response sigma factor SigB prevents competence development in Bacillus subtilis. mBio **15**, e0227424. (10.1128/mbio.02274-24) 39470193 PMC11633097

[183] Brown KL, Hughes KT. 1995 The role of anti-sigma factors in gene regulation. Mol. Microbiol. **16**, 397–404. (10.1111/j.1365-2958.1995.tb02405.x) 7565101

[184] Hughes KT, Mathee K. 1998 The anti-sigma factors. Annu. Rev. Microbiol. **52**, 231–286. (10.1146/annurev.micro.52.1.231) 9891799

[185] Treviño-Quintanilla L, Freyre-González J, Martínez-Flores I. 2013 Anti-sigma factors in E. coli: common regulatory mechanisms controlling sigma factors availability. Curr. Genom. **14**, 378–387. (10.2174/1389202911314060007) PMC3861889 24396271

[186] Bouillet S, Bauer TS, Gottesman S. 2024 RpoS and the bacterial general stress response. Microbiol. Mol. Biol. Rev. **88**, e0015122. (10.1128/mmbr.00151-22) 38411096 PMC10966952

[187] Boylan SA, Redfield AR, Price CW. 1993 Transcription factor sigma B of Bacillus subtilis controls a large stationary-phase regulon. J. Bacteriol. **175**, 3957–3963. (10.1128/jb.175.13.3957-3963.1993) 8320211 PMC204823

[188] Guo MS, Gross CA. 2014 Stress-induced remodeling of the bacterial proteome. Curr. Biol. **24**, R424–R434. (10.1016/j.cub.2014.03.023) 24845675 PMC4089988

[189] Cho Y, Lee E, Ahn B, Roe J. 2001 SigB, an RNA polymerase sigma factor required for osmoprotection and proper differentiation of Streptomyces coelicolor. Mol. Microbiol. **42**, 205–214. (10.1046/j.1365-2958.2001.02622.x) 11679079

[190] Barabási AL, Oltvai ZN. 2004 Network biology: understanding the cell’s functional organization. Nat. Rev. Genet. **5**, 101–113. (10.1038/nrg1272) 14735121

[191] Fang FC, Casadevall A. 2011 Reductionistic and holistic science. Infect. Immun. **79**, 1401–1404. (10.1128/iai.01343-10) 21321076 PMC3067528

[192] Mobus GE, Kalton MC. 2014 Principles of systems science. New York, NY: Springer.

[193] Berkvens A, Chauhan P, Bruggeman FJ. 2022 Integrative biology of persister cell formation: molecular circuitry, phenotypic diversification and fitness effects. J. R. Soc. Interface **19**, 20220129. (10.1098/rsif.2022.0129) 36099930 PMC9470271

[194] Tashiro Y, Kawata K, Taniuchi A, Kakinuma K, May T, Okabe S. 2012 RelE-mediated dormancy is enhanced at high cell density in Escherichia coli. J. Bacteriol. **194**, 1169–1176. (10.1128/jb.06628-11) 22210768 PMC3294780

[195] Evans AC. 1929 Life cycles in bacteria. J. Bacteriol. **17**, 63–77. (10.1128/jb.17.2.63-77.1929) 16559356 PMC375044

[196] Jørgensen MG, Pandey DP, Jaskolska M, Gerdes K. 2009 HicA of Escherichia coli defines a novel family of translation-independent mRNA interferases in bacteria and archaea. J. Bacteriol. **191**, 1191–1199. (10.1128/jb.01013-08) 19060138 PMC2631989

[197] Lohnis F. 1922 Life-cycles of bacteria. Nature **109**, 252–253. (10.1038/109252b0)

[198] Hauryliuk V, Atkinson GC, Murakami KS, Tenson T, Gerdes K. 2015 Recent functional insights into the role of (p)ppGpp in bacterial physiology. Nat. Rev. Microbiol. **13**, 298–309. (10.1038/nrmicro3448) 25853779 PMC4659695

[199] Williams JJ, Hergenrother PJ. 2013 Detection of endogenous MazF enzymatic activity in Staphylococcus aureus. Anal. Biochem. **443**, 81–87. (10.1016/j.ab.2013.08.018) 23994560 PMC3828647

[200] Brereton JLG. 1962 Evolved regulatory mechanisms of population control. In The evolution of living organisms, pp. 81–93. Melbourne, Australia: G.W. Lepar.

[201] Goeders N, Van Melderen L. 2014 Toxin-antitoxin systems as multilevel interaction systems. Toxins **6**, 304–324. (10.3390/toxins6010304) 24434905 PMC3920263

[202] Handler S, Kirkpatrick CL. 2024 New layers of regulation of the general stress response sigma factor RpoS. Front. Microbiol. **15**, 1363955. (10.3389/fmicb.2024.1363955) 38505546 PMC10948607

